# ITGA6 is directly regulated by hypoxia-inducible factors and enriches for cancer stem cell activity and invasion in metastatic breast cancer models

**DOI:** 10.1186/s12943-016-0510-x

**Published:** 2016-03-22

**Authors:** Danielle L. Peacock Brooks, Luciana P. Schwab, Raisa Krutilina, Deanna N. Parke, Aarti Sethuraman, David Hoogewijs, Alexandra Schörg, Lauren Gotwald, Meiyun Fan, Roland H. Wenger, Tiffany N. Seagroves

**Affiliations:** Center for Cancer Research and the Department of Pathology and Laboratory Medicine, The University of Tennessee Health Science Center, Memphis, TN 38163 USA; Institute of Physiology and Zürich Center for Integrative Human Physiology, University of Zürich, CH-8057 Zürich, Switzerland; Present address: National Cancer Institute, Center for Cancer Research, Women’s Malignancies Branch, Bethesda, MD 20892 USA; Present address: Institute of Physiology, University of Duisburg-Essen, 45122 Essen, Germany

**Keywords:** Hypoxia, Hypoxia-inducible factor (HIF), Breast cancer, CD49f, Cancer stem cells (CSC), Invasion, Metastasis

## Abstract

**Background:**

Hypoxia-inducible factors (HIFs) are well-established mediators of tumor growth, the epithelial to mesenchymal transition (EMT) and metastasis. In several types of solid tumors, including breast cancers, the HIFs play a critical role in maintaining cancer stem cell (CSC) activity. Thus, we hypothesized that HIFs may also regulate transcription of markers of breast CSC activity. One approach to enrich for breast cells with stem-like phenotypes is FACS sorting, in which sub-populations of live cells are gated based on the expression of cell surface antigens, including various integrin subunits. Integrin alpha 6 (ITGA6; CD49f) is routinely used in combination with other integrin subunits to enrich for breast stem cells by FACS. Integrins not only mediate interactions with the extracellular matrix (ECM), but also drive intracellular signaling events that communicate from the tumor microenvironment to inside of the tumor cell to alter phenotypes including migration and invasion.

**Methods:**

We used two models of metastatic breast cancer (MBC), polyoma middle T (MMTV-PyMT) and MDA-MB-231 cells, to compare the expression of ITGA6 in wild type and knockout (KO) or knockdown cells. Chromatin immunoprecipitation (ChIP) and luciferase reporter assays verified that ITGA6 is a direct HIF transcriptional target. We also used FACS sorting to enrich for CD49f ^+^ cells to compare tumorsphere formation, tumor initiating cell activity, invasion and HIF activity relative to CD49f^neg or low^ cells. Knockdown of *ITGA6* significantly reduced invasion, whereas re-expression of ITGA6 in the context of HIF knockdown partially rescued invasion. A search of public databases also revealed that ITGA6 expression is an independent prognostic factor of survival in breast cancer patients.

**Results:**

We report that ITGA6 is a HIF-dependent target gene and that high ITGA6 expression enhances invasion and tumor-initiating cell activities in models of MBC. Moreover, cells that express high levels of ITGA6 are enriched for HIF-1α expression and the expression of HIF-dependent target genes.

**Conclusions:**

Our data suggest that HIF-dependent regulation of ITGA6 is one mechanism by which sorting for CD49f ^+^ cells enhances CSC and metastatic phenotypes in breast cancers. Our results are particularly relevant to basal-like breast cancers which express higher levels of the HIFα subunits, core HIF-dependent target genes and ITGA6 relative to other molecular subtypes.

**Electronic supplementary material:**

The online version of this article (doi:10.1186/s12943-016-0510-x) contains supplementary material, which is available to authorized users.

## Background

The rate of rapidly dividing cancer cells in solid tumors quickly surpasses the rate at which new functional blood vessels are formed. In these nutrient- and oxygen-depleted areas, a hypoxic transcriptional response is orchestrated by the Hypoxia-Inducible Factor transcription factors (HIFs), which mediate transcription of multiple genes necessary to adapt to an adverse tumor microenvironment [1]. Most solid tumors overexpress HIF-1α and/or HIF-2α, and over-expression of HIF-1α independently positively correlates with poor prognosis and relapse of breast cancer patients, as reviewed in [2]. We have shown using the mouse mammary tumor virus (MMTV) driven- polyoma virus middle T transgenic mouse model (PyMT) of metastatic breast cancer (MBC) that conditional deletion of *Hif1a* delays onset of palpable tumors, and reduces primary tumor growth rate, lung colonization and overall metastatic burden [3]. Moreover, deletion of *Hif1a* reduces tumor-initiating cell (TIC) frequency and activity in vivo [3]. Therefore, HIF-1 regulates breast tumor growth and metastasis in part by modulating pathways that promote cancer stem cell (CSC)-like activities.

The CSC hypothesis postulates that tumors arise from a small population of cancer cells with stem cell-like properties [4], with a corollary that CSC-like cells play a primary role in relapse due to therapeutic resistance and/or enhanced metastatic potential [5]. Several laboratories have shown that the HIFs play a fundamental role in maintaining CSC potential or a CSC niche in gliomas, neuroblastomas, breast cancers, and hematological malignancies [3, 6–8]. A common feature of hypoxic cells and CSC-like cells is that they are highly refractory to radiation and chemotherapy [9, 10]. For example, hypoxic regions of breast tumors that reappear after treatment of the primary tumor with anti-angiogeneic therapies are enriched with CSC-like cells [11]. Because the HIFs are critical for maintaining CSC/TIC activity in a variety of solid tumors, we hypothesized that HIFs may also regulate transcription of markers used to enrich for CSC-like cells.

Antibodies to integrin subunits that function as heterodimeric receptors for extracellular matrix (ECM) proteins are routinely employed to enrich for normal mammary stem cells and breast CSCs by fluorescence activated cell sorting (FACS). These include integrin beta 1 (ITGB1; CD29), integrin beta 3 (ITGB3; CD61) and integrin α6 (ITGA6; CD49f) [12]. For example, either the CD49f^+^/CD24^+^ [13] or the CD49f^+^/EpCAM^+^ (epithelial cell adhesion molecule) [14] sub-populations will enrich for cells with luminal progenitor potential. In contrast, the CD49f^High^/CD24^−^ sub-population is enriched for basal/mesenchymal phenotypes [14, 15]. Relative to the normal breast tissue, the CD49f^High^/EpCAM^+^ sub-population is enriched in tumors and is believed to mark the lineage that is the origin of luminal breast cancers [15, 16].

Integrins not only mediate interactions with the ECM, but also drive intracellular signaling events that communicate from the tumor microenvironment to inside of the tumor cell to alter migration and invasion. CD49f dimerizes with integrin ß1 or ß4 (ITGB4; CD104) to form either α6ß1 or α6ß4 heterodimers, which bind to laminin, an abundant component of the breast ECM. In the normal breast, α6ß1 is expressed in both the luminal epithelium and myoepithelial cells, whereas α6ß4 is expressed in the myoepithelial cells [17]. Both ß1 and ß4 are implicated in modulating breast tumorigenesis and metastasis [17–19]. In MDA-MB-435 cells, survival under hypoxic stress and metastatic potential depends on expression of the α6ß1 integrin and HIF-1-dependent secretion of VEGF [20], which is a direct HIF target gene. The α6ß4 heterodimer has been shown to mediate cancer cell motility and metastasis [21]. Breast CSC activity was recently shown to depend upon which cytoplasmic domain splice isoform of ITGA6 (α6_A_ or α6_B_) dimerizes with integrin ß1. Cells possessing CSC activity, which also have mesenchymal features, were found to express α6_B_ß1 [22].

A direct contribution of ITGA6 to breast CSC or TIC potential was shown in mammospheres derived from MCF-7 cells, which express higher levels of CD49f relative to bulk cells; knockdown of *ITGA6* also blocked tumor growth in vivo [23]. Relative to the normal mammary gland, expression of *ITGA6* is up-regulated ~4.0-fold in MMTV-Neu tumors [24]. Increased CD49f immunoreactivity in tumor specimens correlates with reduced survival of breast cancer patients [25]. CD49f also enriches for glioblastoma CSCs [26]. Despite accumulating evidence that higher CD49f expression correlates with CSC activity and decreased survival in several cancer types, little is known about how *ITGA6* gene expression is regulated. Since *ITGA6* expression and a HIF transcriptional core gene signature are enriched in basal-like breast tumors relative to luminal (ER+) tumors [15, 27–30], we sought to determine if *ITGA6* might also be a direct HIF target gene.

Herein, we demonstrate that *ITGA6* is a direct transcriptional target of the HIF transcription factors. ITGA6 expression decreases at the mRNA and protein levels in HIF-1 knockout (KO) PyMT cells, or in response to knockdown of both *HIF1A* and *HIF2A* in MDA-MB-231 cells. Three putative hypoxia response elements (HREs) were identified in the *ITGA6* promoter, two of which efficiently bind either HIF-1α or HIF-2α. Enriching for CD49f^+^ PyMT cells enhanced TIC potential as assayed by limiting dilution transplantation. Likewise, enriching for a CD49f^High^ sub-population in MDA-MB-231 cells potentiated cell invasion through Matrigel and HIF-dependent gene expression. The reduced tumor-initiating and invasive potential in the CD49f^Neg/Low^ PyMT or MDA-MB-231 cells overlaps with phenotypes in *Hif1a* null PyMT tumors [3] or *HIF1A* knockdown in MDA-MB-231 tumor cells [31]. Together, these results suggest that HIF-dependent transcriptional regulation of ITGA6/CD49f contributes to the HIFs promotion of TIC and invasion activity.

## Results

### Deletion of HIF reduces ITGA6 expression in PyMT tumor cells

**Fig. 1:**
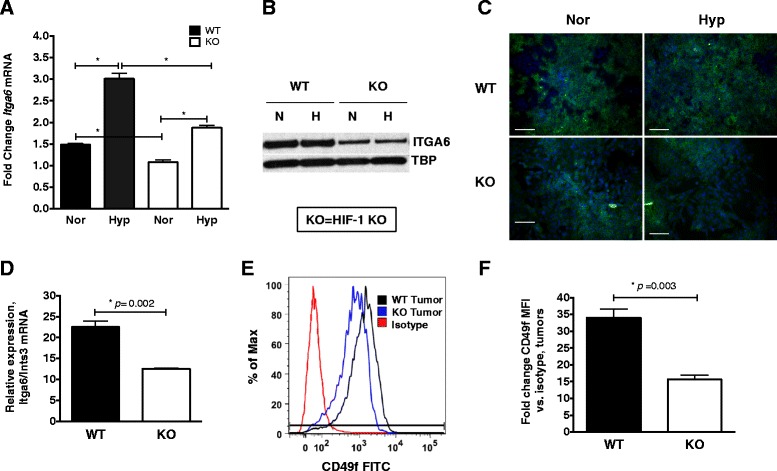
*Itga6* expression is down-regulated in response to deletion of HIF-1 in PyMT+ cultured cells and in tumors. **a** HIF-1 WT or KO cells were cultured at normoxia or hypoxia (6 h, 0.5 % O_2_) and the relative expression of *Itga6* mRNA levels compared after normalization to *Ints3*. The mean fold-change in *Itga6* levels was compared to KO cells cultured at hypoxia (set to 1.0) (*p < 0.01, ANOVA with Bonferroni post-test; *n* = 3 biological replicates). **b** Western blotting was performed for ITGA6 protein using whole cell extracts (WCE) prepared from near confluent HIF-1 WT and KO cells cultured at either normoxia (N) or hypoxia (H). Tata-binding protein (TBP) is shown as a loading control. **c** HIF-1 WT and KO adherent cells were exposed to normoxia or hypoxia (6 h, 0.5 % O_2_), fixed, immunostained with CD49f-FITC and counterstained with DAPI; the scale bar represents 20 μm. **d** The relative abundance of *Itga6* mRNA after normalization to *Ints3* in the same set of HIF-1α WT and KO tumors as subjected to FACS analysis in panel (**e**) (*p* < 0.01, Student’s *t*-test). **e** Representative histograms derived from FACS analysis of live, CD49-FITC stained cells isolated from HIF-1 WT (black histogram) and KO (blue histogram) tumors relative to the isotype antibody control (red histogram). To normalize the histogram height between samples, the y-axis shows the % Max (the number of cells in each bin divided by the number of cells in the bin that contains the largest number of cells). Data shown are representative of n ≥5 tumors/genotype. The percentage of tumor cells positive for CD49f-FITC was determined based on the live, singlet, Lin^neg^ parent population using FlowJo and the gating strategy presented in Additional file 4: Figure S4. **f** The mean ± SEM in the fold change of CD49f-FITC median fluorescence intensity (MFI) between WT and KO PyMT tumors; all data are expressed relative to each genotype’s corresponding isotype control MFI (*p* < 0.01, Student’s *t*-test; n ≥5 tumors/genotype)

*Itga6* mRNA levels were compared in HIF-1 wild type (WT) and knockout (KO) PyMT tumor cells cultured at normoxia or hypoxia by quantitative real-time PCR (qRT-PCR). *Itga6* mRNA levels increased 2-fold in hypoxic WT cells relative to normoxic WT cells, whereas *Itga6* mRNA levels were decreased by ~50 % in KO cells relative to WT cells at hypoxia (Fig. 1a). Western blotting of whole cell extracts (WCE) confirmed that ITGA6 levels were decreased in HIF-1 KO cells relative to WT cells, independent of oxygen tension (Fig. 1b). These results are consistent with our previous observations that PyMT tumor cells express detectable levels of HIF-1α protein at normoxia, although maximal levels of HIF-1α protein accumulate at 6 h of hypoxia (0.5 % O_2_) [3]. Similarly, CD49f-FITC immunofluorescence (IF) staining of fixed PyMT cells revealed a decrease in the total number of CD49f + HIF-1 KO cells relative to WT cells. No striking differences in CD49f staining intensity were observed between normoxia and hypoxic conditions for either genotype (Fig. 1c).

To confirm that CD49f is differentially expressed in vivo, PyMT HIF-1 WT or KO cells were implanted into the mammary fat pad of syngeneic FVB female recipients to generate tumors. Mean *Itga6* mRNA levels were reduced by ~50 % in KO tumors relative to WT (Fig. 1d). When tumors were digested and single cells stained with CD49f-FITC and subjected to FACS profiling (n ≥5 tumors/genotype), the total percentage of CD49f^+^ cells was decreased in HIF-1 KO tumors (Fig. 1e). The average CD49f-FITC median fluorescence intensity (MFI) was also reduced ~1.5-fold in KO tumors relative to WT (Fig. 1f). These results suggested that HIF-1 regulates *ITGA6* gene expression and that the relative changes in mRNA abundance are generally conserved at the protein level.

### Both HIF-1 and HIF-2 regulate expression of ITGA6 in MDA-MB-231 breast cancer cells

**Fig. 2:**
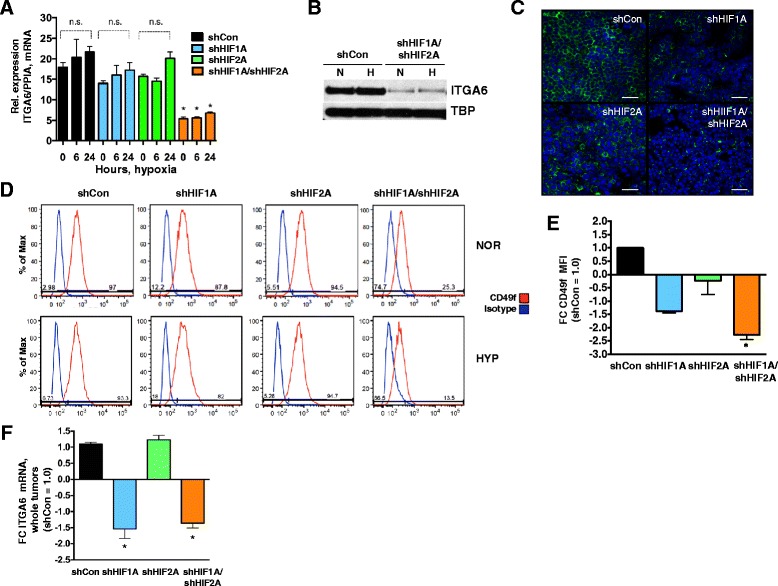
ITGA6/CD49f expression is reduced in response to loss of HIF activity in MDA-MB-231 cells and tumors. **a** Mean ± SEM of the expression of *ITGA6* mRNA relative to *PPIA,* in adherent, cultured MDA-MB-231 cells (*n* = 3 independent biological experiments/genotype). mRNA levels were evaluated after 0, 6 or 24 h of hypoxic exposure. All data are expressed relative to shControl cells cultured at normoxia (set to 1.0); *p-value <0.05 by ANOVA. **b** Western blotting was performed for ITGA6 protein using whole cell extracts (WCE) prepared from near confluent MDA-MB-231 shControl or shHIF1A/shHIF2A cells cultured at either normoxia (N) or chronic hypoxia (H). Tata-binding protein (TBP) is shown as a loading control. **c** MDA-MB-231 shControl, shHIF1A, shHIF2A and shHIF1A/shHIF2A cells were harvested from normoxic cell culture at 90 % confluence, stained with CD49f-FITC in suspension, replated onto chamber well slides and stained with Hoechst 33342; scale bar represents 20 μM. **d** MDA-MB-231 shControl, shHIF1A, shHIF2A and shHIF1A/shHIF2A cells were harvested from normoxic or chronic hypoxic cell culture at 90 % confluence, stained with CD49f-FITC in suspension in flow buffer, and subjected to FACS analysis. Plots represent the % Max of stained cells (*red histogram*) versus the corresponding genotype’s isotype control (*blue histogram*); data presented are representative of 4 biological replicate experiments. **e** The average ± SEM in the fold change (FC) in the CD49f MFI among all four genotypes of cultured cells (*n* = 4 independent experiments/genotype; shControl set to 1.0; *p *<0.05.). **f** The average ± SEM in the fold change (FC) of *ITGA6* mRNA levels expressed in tumors among all four genotypes (*n* = 4 tumors/genotype; shControl set to 1.0; *p* <0.05.); total RNA was prepared from homogenized whole tumors

We next investigated whether HIFs also regulate ITGA6 levels in a MBC cell line of human origin, MDA-MB-231. The MDA-MB-231 cell line models basal/mesenchymal breast cancer, and by molecular profiling is classified as a basal B and claudin-lo subtype [15, 32–34]. MDA-MB-231 cells have been characterized as CD49f^+^/CD24^Neg^ and by FACS up to 99 % of cells are CD49f ^+^ with a 2-log_10_ range of expression levels [15]. In MDA-MB-231 cells, high levels of HIF-1α protein are present at normoxia, and there are modest effects of hypoxic exposure on HIF-1 α protein levels; in contrast, expression of HIF-2α is strongly hypoxia-inducible (Additional file 1: Figure S1c). The effect of shRNA-mediated down-regulation of *HIF1A* alone, *HIF2A* alone or both HIFα subunits (shHIF1A/shHIF2A) on *ITGA6* mRNA levels was compared relative to empty vector transduced cells (referred herein as shControl) cultured at normoxia or hypoxia (0.5 % O_2_; 6 h, “acute” or 24 h, “chronic”). Hypoxic exposure did not significantly increase *ITGA6* mRNA expression levels in shControl cells (Fig. 2a). Likewise, deletion of either *HIF1A* (shHIF1A) or *HIF2A* (shHIF2A) alone was not sufficient to significantly reduce *ITGA6* levels relative to shControl cells. However, when *HIF1A* and *HIF2A* were simultaneously knocked down, a 2- to 3-fold decrease in *ITGA6* expression was observed (shHIF1A/shHIF2A, Fig. 2a). As previously reported, we observed that hypoxic exposure represses transcription of *HIF1A*, but stimulates transcription of *HIF2A* [35] (Additional file 1: Figure S1a, b). shRNA knockdown was not 100 % efficient for either gene as we previously described in [36]. Detectable levels of HIF-1α or HIF-2 α protein were observed in shHIF1A, shHIF2A and shHIF1A/shHIF2A cells, and individual gene knockdown was generally less efficient under hypoxic stress than during normoxic culture (Additional file 1: Figure 1c).

Changes in *ITGA6* mRNA expression were next verified at the protein level. By western blotting, ITGA6 protein levels were reduced in shHIF1A/shHIF2A cells relative to shControl cells in a HIF-dependent, but hypoxia-independent manner (Fig. 2b). IF staining of MDA-MB-231 adherent cells revealed that almost all shControl cells express CD49f. In contrast, there was a reduction in CD49f-FITC staining in both shHIF1A and shHIF1A/shHIF2A cells relative to shControl cells, whereas more subtle reductions in CD49f intensity were noted for shHIF2A cells (Fig. 2c). In agreement with western blot data, by FACS analysis, the percentage of CD49f-FITC^+^ cells present in each genotype of MDA-MB-231 cells did not significantly change in response to hypoxic exposure (Fig. 2d). FACS analysis revealed no statistically significant differences in the total percentage of CD49f ^+^ cells in response to either *HIF1*A or *HIF2A* deletion, although there was general trend of fewer CD49f ^+^ cells in the shHIF1A genotype that was not observed for shHIF2A cells. Independent of oxygen tension, the shHIF1A/shHIF2A cells consistently exhibited a ~3-to-4-fold decrease in the total percentage of CD49f^+^ cells relative to shControl cells (Fig. 2d).

Comparison of the average CD49f MFI among 5 independent FACS experiments revealed that the fluorescent intensity was only significantly reduced when both HIFs were knocked down (Fig. 2e, ~2.3 fold), although there was also trend for a reduction in the MFI in shHIF1A, but not shHIF2A, cells. Together, these results demonstrate that CD49f expression is more dependent upon HIF activity than oxygen tension per se. A similar reduction in percentage of cells staining positive for CD49f-FITC in response to deletion of both HIFα subunits was also observed in MCF-7 cells, a luminal model of breast cancer in which ~10 % of all cells express CD49f [15] (~3-fold decrease for shControl vs. DKD cells; Additional file 2: Figure S2). Finally, qRT-PCR analysis of shControl, shHIF1A, shHIF2A or shHIF1A/shHIF2A MDA-MB-231 whole tumors generated in [36] revealed that *ITGA6* levels were significantly decreased upon knockdown of either *HIF1A* or both HIFα subunits. However, there was no change in *ITGA6* expression in shHIF2A tumors (Fig. 2f). These data suggest that HIF-1 may be predominantly responsible for ITGA6 expression and that HIF-2 can compensate for loss of HIF-1 activity.

### *ITGA6* is a direct HIF transcriptional target gene

**Fig. 3:**
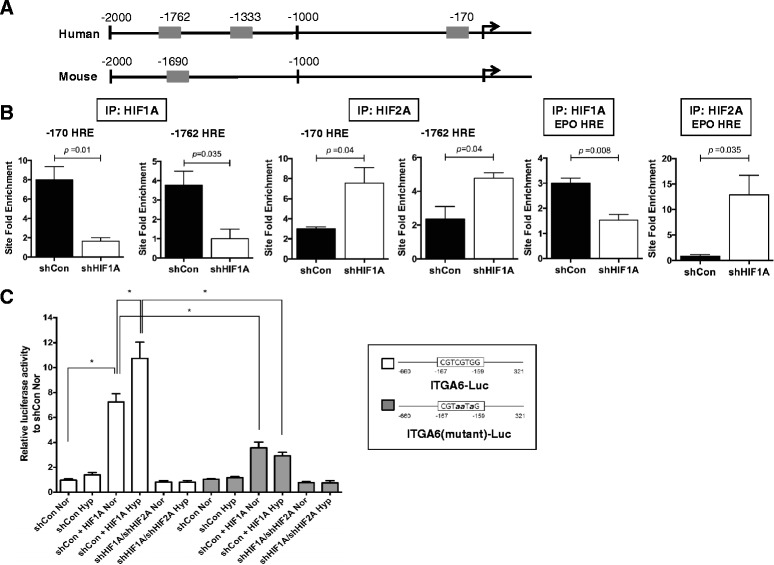
*ITGA6* is a direct HIF transcriptional target gene. **a** A schematic representation of putative HREs identified in the proximal promoter of *ITGA6* that were assessed for HIF-1α and/or HIF-2α recruitment by ChIP assays. **b** MDA-MB-231 shControl and shHIF1A cells were cultured at hypoxia (0.5 % O_2_) for 6 h, and chromatin fragments were immunoprecipitated using HIF-1α or HIF-2α antibodies or anti-rabbit IgG (as the non-specific binding control). SYBR Green-based qRT-PCR was conducted on the purified, isolated DNA fragments to determine the site fold enrichment of HIFα recruitment relative to signal detected in the anti-rabbit IgG control per genotype (qRT-PCR values observed for the IgG control were set to 1.0 per genotype). As the positive control, qRT-PCR was also performed using primers flanking a previously validated, functional HRE site in the 3’ *EPO* enhancer. Each panel shows the mean site fold enrichment ± SEM of technical replicates; data presented are representative of three replicate experiments. **c** Luciferase reporter assays were used to compare relative luciferase activity between MDA-MB-231 shControl or shHIF1A/shHIF2A cells transiently transfected with a wild type ITGA6 promoter linked to luciferase (ITGA6-Luc; white bars) or a HRE mutant promoter construct [ITGA6 (mutant)-Luc; grey bars] and then cultured at normoxia (Nor) or hypoxia (Hyp). In some cases, a stabilized version of murine HIF-1α was also co-transfected (+HIF1A). The mean ± standard deviation are shown; *p* <0.05 by one-way ANOVA followed by Bonferroni's multiple comparison test. The mutant *ITGA6* promoter contains three point mutations within the HIF consensus site

Based on the observed HIF-dependent regulation of *ITGA6* mRNA levels and of CD49f cell surface expression in PyMT, MDA-MB-231 and MCF-7 tumor cells, we next investigated whether *ITGA6* is a direct HIF transcriptional target. Three consensus putative hypoxia response elements (HREs) were identified in the human *ITGA6* promoter (Fig. 3a), located at −170, −1333, and −1762 from the transcriptional start site. A presumably conserved HRE site was also identified in the murine *Itga6* promoter at −1690.

Chromatin immunoprecipitation (ChIP) assays were performed using MDA-MB-231 shControl or shHIF1A knockdown cells (to control for HIF-1α antibody specificity). All cells used for ChIP analysis were cultured at acute hypoxia (6 h, 0.5 % O_2_) since HIF-1α protein levels peak at this time point [36]. HIF-1α was recruited to both the −170 and −1762 HRE sites in shControl cells (Fig. 3b), with a mean site fold enrichment of 4.2-fold or 3.8-fold, respectively (Fig. 3b). A previously validated functional HRE present in the 3’ UTR of the *EPO* enhancer [37] served as the assay positive control (Fig. 3b). In contrast, there was weak enrichment of HIF-1α at the −1333 site in shControl cells relative to shHIF1A cells (Additional file 3: Figure S3A). There was no significant difference in enrichment of either HIF-1α or HIF-2α to a non-HRE site in the promoter (Additional file 3: Figure S3B). Enrichment of HIF-1α at the −1690 site of the murine *Itga6* promoter in PyMT WT cells relative to HIF-1 KO cells was also confirmed (Additional file 3: Figure S3C).

Since we previously reported that HIF-2α protein expression increases in response to *HIF1A* shRNA-mediated gene knockdown in both MCF-7 cells [38] and MDA-MB-231 cells [36], we next investigated if HIF-2α expression could compensate for loss of HIF-1α to regulate *ITGA6* transcription in MDA-MB-231 cells. ChIP assays were repeated for the same HRE sites using anti-HIF-2α antibodies. HIF-2α bound more efficiently to the −170 and the −1762 sites in shHIF1A cells relative to shControl cells (Fig. 3b). Likewise, HRE site enrichment in the EPO 3’ UTR was increased in shHIF1A cells following IP with HIF-2α (Fig. 3b). These results demonstrate that, in MDA-MB-231 cells, either HIF-1α or HIF-2α can potentiate *ITGA6* transcription, as suggested by the expression data presented in Fig. 2.

To confirm the functionality of bound HIF-α subunits to the *ITGA6* promoter, luciferase (Luc) reporter assays were employed. An ITGA6-Luc reporter vector was purchased from SwitchGear that contained the most proximal HRE site validated by ChIP assays, and the HRE consensus site was mutated (ITGA6-mutant-Luc). Relative luciferase activity was measured in the presence or absence of ectopic expression of a stabilized murine HIF-1α mutant that cannot be degraded by the proteasome [39]. Although changes in reporter activity were not observed in response to the endogenous changes in HIFα levels between shControl and shHIF1A/shHIF2A MDA-MB-231 cells, in the presence of transfected mHIF-1α stabilized protein, luciferase activity increased 7.6-fold at normoxia and 10.8-fold at hypoxia relative to shControl normoxic cells (Fig. 3c). Luciferase reporter activity significantly decreased in ITGA6-mutant-Luc transfected cells in the presence of ectopic mHIF-1α.

### CD49f enrichment enhances sphere formation efficiency and TIC activity in the PyMT model

To enrich for CD49f-FITC^+^ cells in PyMT tumors, individual tumors from transgenic females were combined and digested to obtain a single cell preparation. Live, singlet, Lin^neg^/CD31^neg^ tumor cells were sorted based on expression of CD49f and CD24. As we previously described, virtually all cells derived from late stage PyMT carcinomas express CD24 [3]. CD49f^+^/CD24^High^ or CD49f^Neg^/CD24^Low^ cells were enriched in a two-way sort and purity was confirmed by post-sort analysis (refer to Additional file 4: Figure S4).

**Fig. 4:**
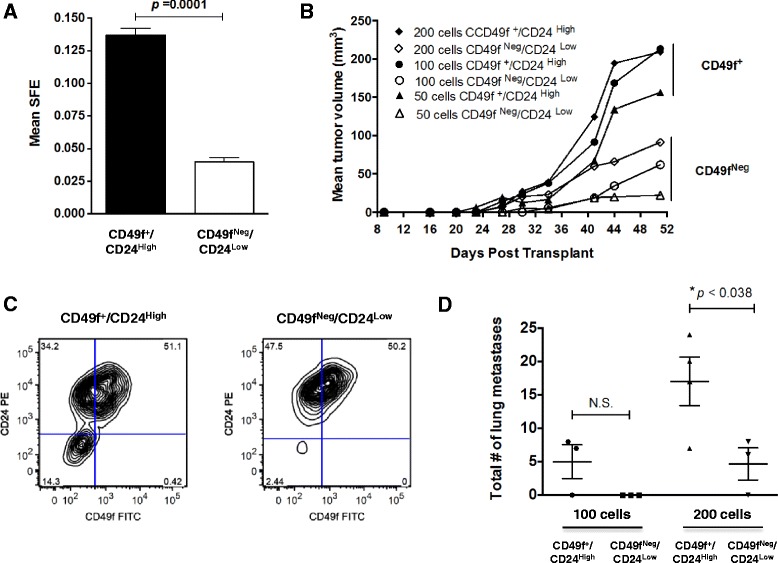
CD49f enriches for TIC activity in vitro and in vivo. **a** Tumors harvested from MMTV-PyMT+ females were harvest, digested, and stained with CD49f-FITC and CD24-PE antibodies prior to FACS sorting. CD49f^+^/CD24^High^ and CD49f^Neg^/CD24^Low^ sorted cell populations were plated in sphere culture conditions to assay for sphere formation efficiency (SFE), or were transplanted by limiting dilution transplantation into female FVB/Nj recipients. **a** Each sorted cell population was plated at a density of 30,000 cells/well in 6-well format and the grand mean SFE ± SEM determined (*n* ≥ 8 wells per genotype; *n* = 3 independent experiments). The *p*-value was calculated by an unpaired Student’s *t*-test. **b** Comparison of changes in mean tumor volume over time when 50, 100 or 200 CD49f^+^/CD24^High^ or CD49f^Neg^/CD24^Low^ sorted cells are transplanted into the cleared inguinal mammary fat pad to assay for TIC potential. The number of tumors that formed for each cohort is indicated in Table 1. **c** CD49f/CD24 expression was analyzed by FACS analysis of end-stage tumors derived from the transplantation of CD49f^+^/CD24^High^ or CD49f^Neg^/CD24^Low^ sorted cells; data are representative of *n* ≥ 4 tumors/transplanted population. **d** Lung metastasis was evaluated in mice bearing tumors originating from CD49f^+^/CD24^High^ or CD49f^Neg^/CD24^Low^ cell populations. A subset of mice in the 100-cell and 200-cell inputs were housed until tumors grew to a volume of ~500 mm^3^ at which time the tumors and lungs were harvested. The mean total number of lung metastases ± SEM present in H&E-stained paraffin sections is shown in the box-and-whisker plot; *p <0.05 by Student’s *t*-test; N.S. equals not significant

Sorted cells were immediately plated into either tumorsphere formation assays in vitro or directly injected into recipient FVB females in a limiting dilution transplantation assay. The sphere formation efficiency (SFE) of CD49f^+^/CD24^High^ cells was ~3.5 times higher than observed of CD49f^Neg^/CD24^Low^ cells (Fig. 4a, *p* = 0.0001; figure representative of three experiments). CD49f^+^/CD24^High^ cells also regenerated primary tumors that were larger in volume at study endpoint than tumors derived from CD49f^Neg^/CD24^Low^ cells, regardless of initial input cell number (Fig. 4b; n ≥ 6 recipient mice/sub-population/cell density, also refer to Table 1). Overall, few palpable tumors were generated by the CD49f^Neg^/CD24^Low^ population (Fig. 4b; *n* = 2 tumors, 200 cells input; *n* = 1 tumor, 100 cells input; *n* = 1 tumor, 50 cells input; *n* = 0 tumors, 25 cells input). When TIC frequency was compared by Extreme Limiting Dilution Analysis (ELDA) software [40] at day 51 post-transplant, the frequency of TICs in the CD49f^+^/CD24^High^ population was significantly higher than in the CD49f^Neg^/CD24^Low^ population- 1 in 99 cells versus 1 in 578 cells, respectively (*p* = 0.00043) (Table 1), an overall enrichment of 5.8-fold. These data demonstrate that CD49f enriches for TIC potential in vitro and in vivo in the PyMT model.

**Table 1:** ELDA analysis of TIC frequency at day 51 post-transplant for the CD49f^Neg^/CD24^Low^ or CD49f^+^/CD24^High^ sorted cell populations isolated from MMTV-PyMT+ tumors that were implanted into the cleared mammary fat pad of recipient mice at low density; the number of animals producing tumors/the number of animals transplanted is also indicated

	Sorted population:		
Number of cells injected:	CD49f^Neg^/CD24^Low^	CD49f^+^/CD24^High^	*p*-value:
200	2/7 (29 %)	5/7 (71 %)	
100	1/6 (17 %)	4/6 (67 %)	
50	1/8 (12 %)	6/9 (67 %)	
25	0/8 (0 %)	1/8 (12 %)	
Estimated TIC Frequency	1/578	1/99	
(95 % C.I.)	(1/216 to 1/1,544)	(1/58 to 1/170)	0.00043

End-stage primary tumors derived from limiting dilution transplantation were also harvested and digested to generate a single cell preparation for FACS analysis to compare CD49f-FITC and CD24-PE expression in regenerated tumors (Fig. 4c; data representative of n ≥5 tumors/sorted population). We observed that all tumors derived from CD49f^+^/CD24^High^ cells were composed of 3 distinct cell populations: a CD49f^+^/CD24^High^ population (~51 %), a CD49f^Neg^/CD24^High^ population (~34 %), and a population of CD49f^Neg^/CD24^Low^ cells (~14 %). In contrast, none of the tumors derived from CD49f^Neg^/CD24^Low^ cells regenerated a CD49f^Neg^/CD24^Low^ population, with cells almost equally divided between the CD49f^+^/CD24^High^ and the CD49f^Neg^/CD24^High^ quadrants. Therefore, CD49f^+^/CD24^High^ cells can regenerate both the cell population of origin, and a CD49f^Neg^/CD24^Low^ population.

Although these studies were not powered *a priori* to compare lung metastases, metastatic burden was also compared in a subset of mice that were implanted with either 100 or 200 cells input and in which tumors were allowed to grow to a volume of 500 mm^3^. At this tumor volume, the lungs were harvested in conjunction with the primary tumor. When scored for the total number of lung lesions, more metastases were present in mice bearing mammary tumors derived from CD49f^+^/CD24^High^ than CD49f^Neg^/CD24^Low^ cells. These data were significant for the 200-cell cohort, but did not reach statistical significance for the 100-cell cohort (Fig. 4d).

### ITGA6 is necessary for efficient invasion in MDA-MB-231 cells and ectopic expression can partially rescue invasion of shHIF1A/shHIF2A cells

**Fig. 5:**
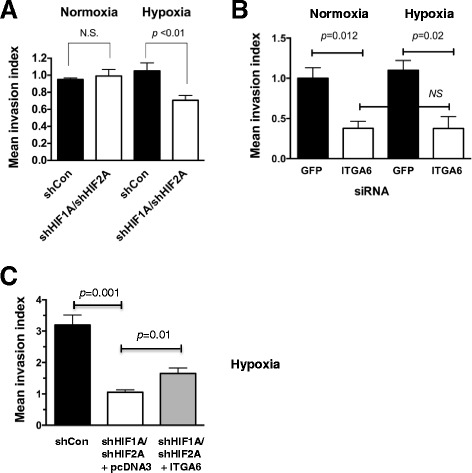
Manipulation of ITGA6 levels directly impacts invasion. **a** Non-sorted, cultured shControl or shHIF1A/shHIF2A MDA-MB-231 cells were evaluated for invasive potential using Boyden chamber assays. The grand mean of the invasion index (invasion corrected for random migration) ± SEM of 3 independent experiments is shown; *p*-values calculated by Student’s *t*-test are indicated. **b** Knockdown of *ITGA6* impairs invasion at normoxia and hypoxia. The grand mean of the invasion index (invasion corrected for random migration) ± SEM of 3 independent experiments is shown; *p*-values were calculated by Student’s *t*-test. **c** Ectopic expression of *ITGA6* in shHIF1A/shHIF2A cells partially rescues invasion at hypoxia. The grand mean of the invasion index (invasion corrected for random migration) ± SEM of 3 independent experiments is shown; *p*-values were calculated by Student’s *t*-test.

It is well-established that the HIFs are required to promote efficient invasion of breast cancer cells through Matrigel in response to hypoxic stress in vitro, and are essential in vivo for efficient lung metastasis from the mammary gland or in tail vein assays [3, 41]. Because CD49f expression is virtually eliminated in shHIF1A/shHIF2A MDA-MB-231 cells, and since either HIF-1 or HIF-2 can regulate transcription of *ITGA6* (Fig. 3), we first compared invasion of non-sorted shControl and shHIF1A/shHIF2A cells cultured at normoxia or hypoxia. No significant changes in the invasion index (corrected for random migration) were observed at normoxia, but invasion decreased by ~33 % in shHIF1A/shHIF2A cells at hypoxia (Fig. 5a).

To determine if invasion requires ITGA6, *ITGA6* mRNA was knocked down by transient transfection with siRNAs in MDA-MB-231 cells; siRNA to GFP served as the control. At normoxia, the mean invasion index was reduced in ITGA6 siRNA cells by 60 %, and at hypoxia, the mean invasion index was reduced by 67 %. In agreement with our observations that ITGA6 expression is dependent upon HIF activity, but not hypoxic stress (Figs. 1 and 2), hypoxic exposure had no effect on invasion of the ITGA6 siRNA cells, as there was no significant difference in invasion between normoxia or hypoxia (Fig. 5b). Since ITGA6 is only one of many HIF-responsive target genes, we next tested whether ectopic expression of ITGA6 in the shHIF1A/shHIF2A context could rescue invasion potential. A stable shHIF1A/shHIF2A cell line was created expressing ITGA6, resulting in a 37 % percent increase in expression relative to shHIF1A/shHIF2A cells (Additional file 5: Figure S5) and invasion was compared at hypoxia. Although the effects on invasion were modest, re-expression of ITGA6 increased invasion in a statistically significant manner relative to shHIF1A/shHIF2A cells (Fig. 5c).

### Enrichment of invasion potential and HIF-dependent gene expression in CD49f^+^ MDA-MB-231 cells

**Fig. 6:**
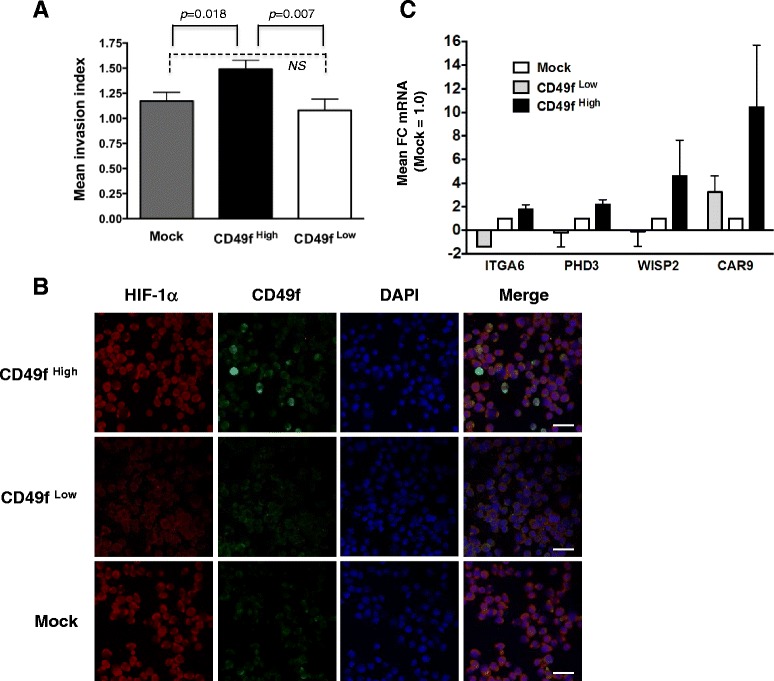
Enriching for CD49f by cell sorting enhances cell invasion in MDA-MB-231 cells. **a** Mock-sorted, CD49f^High^ or CD49f^Low^ MDA-MB-231 cell populations were exposed for 24–48 h to hypoxia and invasion then compared (*n* = 3-4 replicate wells/population/experiment; data are representative of 3 independent sort experiments); *p*-values calculated by Student’s *t*-test are shown. The grand mean invasion index ± SEM is expressed relative to Mock-sorted cells (cells stained with CD49f-FITC but not gated during sort; set to 1.0). **b** IF staining for CD49f-FITC and HIF-1α in cytospun CD49f^High^, CD49f^Low^ or Mock sorted cells counterstained with DAPI; scale bar = 50 μm. **c** qRT-PCR analysis to compare expression levels of known HIF-dependent target genes in the CD49f^High^ vs. CD49f^Low^ cell populations; all values were first normalized for loading and were then normalized to Mock sorted cells (white bars; fold change, FC set to 1.0). The mean ± SEM is reported for three independent experiments

We next investigated whether metastatic potential is enhanced in MDA-MB-231 cells enriched for CD49f expression (CD49f^High^) when cultured at hypoxia. Cultured shControl cells were gated for live, singlet cells and then two-way sorted on the basis of CD49f-FITC levels into either CD49f^High^ or CD49f^Low^ populations, representing the upper or lower 20 % of singlet-gated cells (refer to Additional file 6: Figure S6). Sorted cells were allowed to recover overnight in stem cell media and were then plated for invasion assays. We observed that the invasion index was significantly higher in CD49f^High^ cells relative to either CD49f^Low^ cells or to stained, but mock-sorted cells (Mock; Fig. 6a).

A subset of sorted cells were also cytospun onto slides and stained with HIF-1α antibodies, which revealed an enrichment for HIF-1α protein in CD49f^High^ cells relative to CD49^Low^ cells (Fig. 6b). In contrast, no discernable differences in HIF-1α signal were observed between mock-sorted and CD49f^High^ cells, as might be expected since ≥ 95 % of shControl cells stain with CD49f by FACS analysis (Fig. 2d). The HIF-1α signal was predominantly detected in the cytoplasm of MDA-MB-231 cells, likely because the sorted cells were exposed to ambient oxygen tensions during FACS sorting and during all antibody incubations.

Expression levels of known HIF-dependent target genes were next compared among the Mock-sorted, CD49f^High^ and CD49f^Low^ cell populations by qRT-PCR (Fig. 6c). As expected, *ITGA6* mRNA levels were decreased in CD49f^Low^ cells and enriched in CD49f^High^ cells relative to Mock-sorted cells. Expression of prolyl hydroxylase 3 (PHD3), a gene predominantly regulated by HIF-1 rather than HIF-2 in breast cancer cells [38], was enriched in CD49f^High^ cells by ~2.5-fold. Carbonic anhydrase IX (CAR9), which is also predominantly regulated by HIF-1 in breast cancer cells [38], was up-regulated in CD49f^High^ cells by >11-fold relative to Mock-sorted or CD49f^Low^ cells. Expression of Wnt-1-inducible signaling pathway protein-2 (WISP2), a gene predominantly regulated by HIF-2 than HIF-1 in breast cancer cells [38], was enriched by >5.0 fold in CD49f^High^ cells (Fig. 6c). These data demonstrate that HIF-1α is functional and that hypoxic transcriptional outputs are enriched in CD49f^High^ relative to CD49f^Low^ MDA-MB-231 cells.

### High expression levels of ITGA6 independently correlate with survival of breast cancer patients

**Fig. 7:**
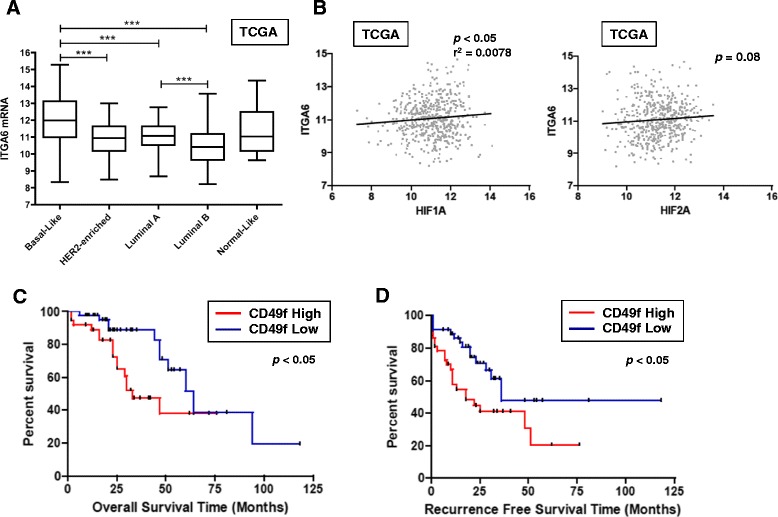
ITGA6 expression levels are prognostic of overall or recurrence-free survival of breast cancer patients. **a** *ITGA6* mRNA is differentially expressed in human breast cancers classified by the PAM50 subtype in the TCGA data set [30]; ***p < 0.001, ANOVA with Bonferroni post-test; (*n* = 93 basal, 56 HER2+, 228 Luminal A, 123 Luminal B and 7 normal-like tumors). **b** *ITGA6* mRNA levels also weakly positively correlate with *HIF1A* levels (*p* <0.05, correlation coefficient *r* ^2^ = 0.0078; TCGA data), but not with *HIF2A* mRNA levels (*p* = 0.08). **c**-**d** Kaplan-Meier curves depict the probability of overall survival (**c**) or recurrence-free survival (**d**) based on relative *ITGA6* mRNA expression from clinical data reported in [42]. *ITGA6* expression levels were stratified by the upper quartile or lowest quartiles (*n* = 37 tumors for “High” CD49f expression, and *n* = 45 tumors for “Low CD49f”) and significance determined by the log-rank test, *p < 0.05)

Breast cancers have been classified into five major subtypes on the basis of global gene expression; one of several genes up-regulated in basal-like cancers relative to other subtypes is *ITGA6* [27]. We first confirmed using The Cancer Genome Atlas (TCGA) data [30] that *ITGA6* expression is enriched in basal-like breast cancers (Fig. 7a). A similar level of enrichment in basal-like cancers was also detected in a second independent dataset [42] [GEO: GSE1992; data not shown]. We next queried if expression of *ITGA6* correlated with *HIF1A* or *HIF2A* mRNA levels in the TCGA dataset. A small, but statistically significant, positive correlation was observed between *HIF1A* and *ITGA6* expression in the TCGA samples (r^2^ 0.0078, *p* < 0.05). In contrast, no correlation was observed between *ITGA6* and *HIF2A* expression (Fig. 7b). When patient samples were stratified by the highest and lowest quartiles of tumor *ITGA6* expression [GEO: GSE1992], higher levels of *ITGA6* expression predicted significantly shorter overall survival (OS; Fig. 7c) and recurrence-free survival (RFS; Fig. 7d) as compared to patients with low *ITGA6* expression.

## Discussion

The molecular networks that promote metastasis downstream of HIF are not yet fully elucidated. Likewise, the mechanisms by which enriching for CD49f selects for cells with stem-like activities in breast or other cancers, including prostate [43] and glioblastoma [26], are poorly understood. We have demonstrated using two models of metastatic breast cancer that *ITGA6* is a HIF-dependent transcriptional target gene. Our data suggest that *ITGA6* is regulated in a HIF-dependent, but oxygen-tension independent manner and that ITGA6 expression can be regulated by HIF-2 when HIF-1α levels are reduced. This mode of regulation is in agreement with our previous observations that the individual HIFα isoforms tend to modulate the kinetics of target gene expression rather than specific sets of gene targets [38]. One caveat of our study is that the shRNAs employed to knockdown *HIF1A* and *HIF2A* did not completely eradicate HIFα protein expression under hypoxic stress, although few shRNAs target with 100 % efficiency. Future studies employing CRISPR/Cas9 mediated gene editing to create efficient knockout lines may be useful to test the requirement for HIF-1 vs. HIF-2 in the regulation of ITGA6 expression.

We propose that ITGA6 functions as one key mediator of the well-characterized HIF-dependent promotion of CSC/TIC and metastatic activities [8, 44]. This is based on our observations using in vivo samples that loss of HIFα subunits reduces *ITGA6* mRNA expression and that enriching for CD49f^+^ in PyMT cells enhances tumorsphere formation, tumor initiation and lung metastasis. Our results support previous studies describing a positive, direct relationship between *ITGA6* levels and metastatic potential using a panel of breast cancer cell lines, including MDA-MB-231 cells [45]. Of note, an inverse relationship between *ITGA6* mRNA and estrogen receptor (ER) mRNA levels was identified in this study, prior to the discovery that *ITGA6* mRNA is a marker of the basal-like breast cancer subtype [27]. Moreover, the presence of EpCAM^-^/CD49f ^+^ cells has recently been shown to increase metastatic potential and to reduce disease-free survival of breast cancer patients [46]; MDA-MB-231 cells are classified as EpCAM^−^/CD49f ^+^ [15].

Increasing evidence supports observations that the relationship between HIF activity and CD49f expression is more prominent in basal breast cancers as compared to luminal cancers. First, a HIF transcriptional core gene signature is significantly enriched in basal breast cancers [29, 30]. Second, we have shown that HIF-1α and HIF-2α protein levels are enriched in basal breast cancers relative to luminal tumors [36]. We also confirm herein that *ITGA6* expression is an independent prognostic factor of RFS and OS in breast cancer patients, as was suggested in [25] and was shown in [46]. Our results are also similar to those of Ali et al., who found that increasing levels of CD49f protein are prognostic of reduced survival, but only in ER negative patients [47].

Several markers routinely used to enrich for breast CSCs by FACS are regulated by hypoxia via HIF-1, including heat stable antigen (CD44) and CD24 [48, 49]. The pairing of CD44 and CD24 was first employed to select for TICs in solid tumors, leading to observations that in breast cancer the CD44^High^/CD24^Neg/Low^ sub-population has enriched TIC activity [50]. In contrast, in several transgenic mouse models of breast cancer, the consensus is that the CD24^+/High^ sub-population rather than CD24^Neg/Low^ sub-population enriches for TIC activities when CD24 is paired with either CD29 (ITGB1) or CD61 (ITGB3) [51–53]. Hypoxia also regulates transcription of *Prominin-1* (PROM1; CD133) [54]. CD133 has been widely employed to enrich for CSCs in several types of solid tumors. In particular, in patient-derived xenografts, when combined with CD49f and CD44, CD133 further enriches for cells with CSC activity, but only in basal-like tumors [55, 56]. We previously identified CD133 as a HIF-dependent gene that, when combined with CD24, enriches for tumorsphere formation in the PyMT model [3]. There are also an increasing number of direct connections between hypoxia and CSC activity. Particularly germane to the oxygen tension fluctuations typical of solid tumors, repeated exposure of breast cancer cells to cycles of normoxic and hypoxic conditions increases the frequency of CSCs, and leads to a more aggressive phenotype [57].

Several studies have shown that enrichment for CD49f^+^ cells enhances, but that loss of function represses, tumorigenesis and metastasis phenotypes. For example, *Itga6* knockdown in 4T1 cells decreases metastasis [58]. In the *Brca1* mutant mouse model, deletion of *Itga6* alone, or along with CD29 significantly repressed metastasis [59], and in the MMTV-Neu model, CD49f paired with CD61 enriches for a sub-population of cells with enhanced TIC potential [58]. In MCF-7 cells, CD49f enrichment is sufficient to select for cells with enhanced stem-like properties, including the ability to form mammospheres in vitro and to more efficiently produce tumors in vivo [23]. In a prostate cancer model, animals with established bone metastasis that were treated with blocking antibodies to CD49f exhibited decreased progression of osteolytic disease [60]. Therefore, high levels of CD49f correlate with enhanced tumorigenesis and metastasis phenotypes. Although fewer MCF-7 cells are positive for CD49f relative to MDA-MB-231 or other mesenchymal-like breast cancer cell lines [15], we observed a conserved decrease in CD49f expression in shHIF1A/shHIF2A MCF-7 cells.

We also find that the CD49f^High^ MDA-MB-231 cells express higher levels of HIF-1α protein and mRNAs of known downstream HIF target genes relative to Mock-sorted or CD49f^Low^ cells. Therefore, we propose that selecting for cells that express high levels of CD49f may also co-select for cells with an enhanced hypoxic response. *CAR9*, a surrogate marker of tumor hypoxia [61] that promotes tumor growth and lung metastasis [62], was highly enriched in CD49f^High^ cells. However, unexpectedly based on the levels of HIF-1α immunostaining observed in the sorted cell populations, *CAR9* expression was also enriched in CD49f^Low^ cells relative to Mock cells. It is possible that differences in HIF-2α protein levels between Mock, CD49f^Low^ and CD49f^High^ cells may also contribute to the gene expression changes we observed in sorted cells since HIF-1 and HIF-2 often compensate for each other to regulate transcription of target genes [38]. CAR9 expression correlates independently with OS in patients with invasive breast cancer [63] and like, CD49f, CAR9 expression is highest in the basal subtype [64]. Moreover, loss of *CAR9* represses breast CSC activities and expression of genes involved in EMT and maintaining stemness [65]. Another HIF-dependent target expressed at higher levels in CD49f^High^ MDA-MB-231 cells was *PHD3*; HIF activity is essential for *PHD3* expression since deletion of HIF-1 and HIF-2 eliminates *PHD3* expression in breast cancer cells [38].

In addition to their utility as an enrichment method to capture cells with CSC activities, integrins are essential for regulating invasion and migration of tumor cells during metastasis [66]. Integrin function is crucial not only for physically tethering cells to the matrix, but also for sending and receiving molecular signals that regulate these processes during tumorigenesis [67]. Integrins are also involved in multiple points of the metastatic cascade, including local invasion and growth in distant organs. Yet, which transcription factors are required for regulation of *ITGA6* gene expression is poorly defined. Functional Sp1/Sp3 sites in the promoter region have been confirmed by ChIP analysis [68], and consensus binding sites for NF-κB, AP-1 and Myc, were also identified [69]. A search of ChIP-seq data deposited into the University of California Santa Cruz (UCSC) Genome Browser by the Encyclopedia of DNA elements (ENCODE) consortia confirmed that Myc, which shares a consensus binding elements with HIF-1, binds to the *ITGA6* promoter [ENCODE: ENCSR000DMQ]. Yet, in MCF-7 cells, a ChIP-seq pipeline to identify HIF-1 and HIF-2 binding sites did not reveal *ITGA6* as a high stringency HIF target [70]. It is possible that there are differences in DNA accessibility for HIF binding sites between MDA-MB-231 and MCF-7 cells, or that the stringency of the ChIP-seq data analysis pipeline described in [70] excluded *ITGA6.* We recently validated breast tumor kinase (*BRK/PTK6*) as a HIF-dependent gene highly expressed in basal breast cancers [36]; however, PTK6 was also not identified as a HIF-dependent gene in this ChIP-seq study [70].

At the post-translational level, chronic hypoxia increases the cell surface localization of CD49f in a Rab11-dependent manner, leading to increased α6ß4 at the cell membrane and increased invasion in MDA-MB-231 cells [71]. In agreement with our results that loss of HIFα function, rather than hypoxic exposure, impacts *ITGA6* transcription, the authors of this study did not report any hypoxia-dependent differences in *ITGA6* mRNA levels. Changes in ECM stiffness are also implicated in promoting breast tumor invasion through the integrins [72]. In fact, in normal mammary epithelial cells, increasing ECM stiffness is sufficient to induce malignant phenotypes, which are sensed in part through a α6ß4, Rac1 and PI3K signaling pathway [73]. Hypoxic exposure is one of many stressors that increase cell stiffness [74]. Hypoxia-induced matrix stiffening can stimulate cell motility [75]. The level of ITGA6 protein was also recently found to increase in myofibroblast cells in response to increased stiffening of a polyacrylamide matrix [76].

In summary, CD49f enrichment is likely to enhance multiple cell motility and cell signaling outputs that crosstalk to promote malignancy and metastatic phenotypes, including CSC-like behaviors. The relationship between high levels of HIFα protein and increased expression of HIF transcriptional targets, including *ITGA6,* in basal breast cancers likely directly contributes to their enhanced aggressive nature, including the enhanced risk of relapse within the first five years of diagnosis [77]. Future studies employing genetic modulation of ITGA6 levels may be useful in identifying druggable targets downstream of ITGA6 to block metastasis.

## Conclusions

We identify *ITGA6* as a novel HIF-dependent target gene that controls stem-like cell phenotypes and tumor cell invasion in pre-clinical models of metastatic breast cancer. Enrichment for CD49f in breast cancer cells also enriches for HIF-1α expression and expression of downstream HIF target genes. Thus, our data suggest that HIF-dependent regulation of the *ITGA6* gene is one mechanism by which sorting for CD49f + cells enhances CSC and metastatic phenotypes in breast cancers since HIF-1 activity is highest in CD49f^High^ cells. These relationships are particularly relevant to basal-like breast cancers which express higher levels of HIF-dependent target genes and *ITGA6* relative to the other common molecular subtypes.

## Methods

### Animals

MMTV-PyMT+ transgenic mice (FVB/Nj strain) were generously provided by Dr. Kent Hunter (National Cancer Institute). FVB/Nj female mice were purchased from The Jackson Laboratory (Bar Harbor, ME, USA). All animal procedures were approved by the Institutional Animal Care and Use Committee at the University of Tennessee Health Science Center in Memphis, TN (UTHSC).

### Cell culture

PyMT+ HIF-1 WT and KO cells were generated and propagated in cell culture as described in [3]. MDA-MB-231 or MCF-7 cells in which *HIF1A* (shHIF1A), *HIF2A* (shHIF2A) or both genes (shHIF1A/shHIF2A) were stably knocked down by shRNA targeting were generated using pLKO.1-based lentiviruses in the Wenger laboratory, and validated for knockdown efficiency in [38]. Cells transduced with empty vector virus (pLKO.1-puro) were used as controls (shControl). All MDA-MB-231 cell lines and tumors xenografted in immunocompromised mice were previously generated and characterized in [36]. MDA-MB-231 cells and MCF-7 cells were authenticated by DDC Medical (Fairfield, OH) and a search of the short tandem repeat (STR) database hosted by ATCC. All human cell lines were grown in DMEM-Hi, supplemented with 10 % FBS, 1× antimycotic-antibiotic (AA) (Sigma, St Louis, MO, USA), 25 mM HEPES and shRNA selection antibiotics as in [36, 38]. Cells were routinely screened for mycoplasma using the MycoAlert kit (Lonza, Basel, Switzerland).

### Antibodies

All antibodies and dilutions used in experiments are listed in Additional file 7: Table S1.

### Flow cytometry and cell sorting

Cells grown in monolayer were detached with 0.25 % trypsin/EDTA and washed several times with HBSS prior to resuspension in flow buffer (HBSS containing 2 % FBS, 10 mM HEPES, and 1 mg/ml DNase I). Cell number and viability were verified by trypan blue staining prior to antibody staining. All cultured cells (1 × 10^6^ in 200 μl flow buffer) were stained on ice for 1 h with CD49f-FITC (cat#555735, BD Biosciences, San Jose, CA). PyMT cells were also co-stained with CD24-PE (cat#553262). Samples were rinsed once with 2 ml flow buffer, pelleted and kept on ice until analysis at the UTHSC Flow Cytometry core on a LSR II flow cytometer. All raw data were exported from FACSDiva software and analyzed using FlowJo v8.8.7 software (Tree Star, Ashland, OR). For each genotype of cells, a representative plot of the percentage of CD49f-FITC^+^ cells (red histogram) relative to the corresponding isotype control (blue histogram) is shown.

FACS analysis of PyMT HIF-1 WT or KO tumors was performed as follows. HIF-1 WT or KO tumors were derived from HIF-1 WT and KO MTECs transplanted into the right inguinal mammary fat pad of 3-week old FVB/Nj female recipients (50,000 cells input) as in [3]. Late stage tumors (350–750 mm^3^ volume) were minced to a fine paste and digested with collagenase type III (Worthington Biochemical Corp, Lakewood, NJ, USA). Single cells from tumors (40 × 10^6^ cells total) were subjected to immunostaining using CD49f and CD24 antibodies. Tumor cells were also stained with the biotin-conjugated mouse lineage panel (cat#559971) and CD31-biotin (cat#553371), followed by streptavidin (SA)-APC (cat#554067) to facilitate gating against hematopoietic lineage (Lin)-positive cells and/or endothelial cells present in whole tumors. Cells were rinsed with flow buffer and then sorted for purity using the 100 μm nozzle on a FACSAria Cell Sorter (BD Biosciences) using the gating strategy described in [3] and also presented in Additional file 4: Figure S4.

After exclusion of dead cells and doublets, PyMT tumor cells were sorted for two cell populations: CD49f^+^/CD24^High^ or CD49f^Neg^/CD24^Low^, whereas MDA-MB-231 cells were sorted only for CD49f^High^ or CD49f^Low^ as these cells are negative for CD24 [15]. The gating strategy for CD49f-FITC sorting of MDA-MB-231 cells is presented in Additional file 6: Figure S6. Mock-sorted MDA-MB-231 or PyMT cells were exposed to primary antibodies, but no gates were applied during sorting. All sorted cells were collected into 4.5 ml tubes pre-coated with FBS and filled with 1 ml of DMEM containing 20 % FBS. Post-sort analysis was performed to verify purity and viability (by 7-AAD) of sorted populations. Cell viability was re-confirmed in the laboratory by trypan blue staining and hemacytometer analysis just prior to downstream assays.

### Immunofluorescence (IF)

PyMT+ HIF-1 WT and KO tumor cells were plated onto tissue-cultured treated, chamber-well slides in standard culture media. At 80 % confluence, cells were placed at normoxia or hypoxia (0.5 % O_2_) for 6 additional hours. Slides were fixed with 4 % paraformaldehyde for 20 min at room temperature followed by immunostaining with anti-CD49f-FITC (1:50, BD Biosciences). For MDA-MB-231 cells, trypsinized cells were stained in suspension in FACS buffer at a dilution of 1:50 CD49f-FITC, washed, counterstained with Hoechst 33342, and cytospun onto glass slides. For MDA-MB-231 sorted cells, each cell population (Mock, CD49f^High^ or CD49f^Low^) was cytospun onto glass slides and fixed with methanol for 20 min. FITC-signal was allowed to fade for 7 days, then slides were re-stained with anti-CD49f-FITC and anti-HIF-1α antibodies, followed by counterstaining with DAPI. All slides were mounted in SlowFade mounting medium and digitally imaged on a Zeiss LSM700 confocal microscope using identical capture times and settings. Un-manipulated raw (.tiff) images are presented.

### Gene expression

Total RNA was extracted from PyMT or MDA-MB-231 parental cultured cells, or Mock-sorted, CD49f^High^ or CD49f^Low^ MDA-MB-231 cell populations sorted from shControl cells, or from pulverized whole tumors using RNABee (Tel-Test, Friendswood, TX). RNA integrity was assessed by the Agilent 2100 Bioanalyzer (Santa Clara, CA, USA) at the UTHSC Molecular Resource Center. Total RNA was transcribed into cDNA using the High-Capacity cDNA reverse Transcription kit (Applied Biosystems, Foster City, CA, USA). Optimized primer and probe sets were designed with the Roche Universal Probe Library assay Design Center software (Roche Applied Science, Indianapolis, IN). *Ints3* (integrator complex subunit 3) or *PPIA* (cyclophilin A) were used as a loading control for murine or human samples, respectively. The advanced relative quantification algorithm or the relative expression quantification algorithm of the Roche LightCycler 480 system was used to calculate gene expression relative to the loading control. Analysis of expression of HIF target genes in MDA-MB-231 sorted cells (*PHD3, CAR9, WISP2*) was performed using LightCycler 480 SYBR Green master mix and the relative expression comparison method after normalization to cyclophilin A (*PPIA*), using the primers reported in [38]. Primer sequences/probes are provided in Additional file 7: Tables S2–S3.

### Promoter analysis and chromatin immunoprecipitation (ChIP) assays

The *Itga6* and *ITGA6* proximal promoters (−2000 to +500 bp) were scanned for the presence of putative functional hypoxia response elements (HREs) using the Transcription Factor Matrix (TFM) Explorer algorithm applying weight matrices available from JASPAR and TRANSFAC. Primers were designed to three independent HREs in the *ITGA6* promoter, or the one HRE site in the *Itga6* (murine) promoter, as well as to non-HRE sequences (Additional file 7: Table S4). MDA-MB-231 shControl or shHIF1A transduced cells were cultured at 21 % (normoxia) or 0.5 % O_2_ (hypoxia) for 6 h and fixed with 1 % formaldehyde for 12 min. DNA was sheared to ~500 bp fragments by sonication using a Diagenode Bioruptor™ set to high, with a 30 s burst followed by 30 s cooling for 10 min. ChIP was performed using primary antibodies against HIF-1α, HIF-2 α or rabbit IgG, the control for non-specific antibody binding. Antibodies were incubated with protein A agarose beads and sheared DNA overnight at 4 °C, washed and eluted in 100 μl elution buffer (1 % SDS plus 0.1 M NaHCO_3_). Reverse crosslinking was accomplished by adding 1 μl of 10 mg/ml RNase and 5 M NaCl to a final concentration of 0.2 M and incubation at 65 °C for 5 h, followed by digestion with Proteinase K at 37 °C for 1 h. Immunoprecipitated DNA was recovered using the ChIP DNA Clean and Concentrator kit (Zymo Research, Irvine, CA). qRT-PCR was performed on all samples using LightCycler 480 SYBR Green master mix. Input DNA (non-IP enriched) values were used to normalize each ChIP DNA fraction’s Cp (crossing point) value to the input DNA fraction Cp value to account for chromatin sample preparation differences (ΔCp_Normalized ChIP_). The normalized ChIP fraction Cp values were then adjusted based on the normalized IgG only IP fraction Cp value (∆∆C*p* = (ΔCp_Normalized ChIP_ - (ΔCp_Normalized IgG_). The ChIP assay site fold enrichment above the sample specific background was then calculated as 2^(−∆∆Cp)^. Previously validated HRE sites present in the erythropoietin (*EPO*) 3’ UTR or the *Vegf* proximal promoter amplicons were used as a positive control. The sequences of the primers that span HRE regions are listed in Additional file 7: Table S4. Data are presented as the mean site fold enrichment ± SEM after correction for input and the IgG control for each genotype of cells (shControl vs. shHIF1A), and are representative of three independent experiments.

### Luciferase reporter assay

A commercial ITGA6 luciferase reporter construct was purchased from SwitchGear Genomics (catalog number S708174). The HRE site contained within this fragment was mutated to generate a ITGA6 (mutant)-Luc reporter using the Phusion Site-Directed Mutagenesis Kit (Thermo Scientific) and the following 5’-phosphorylated primers: Forward 5’-GGGGCTCCCACGTaaTaGCTTCCGGGC-3’ and Reverse 5’-GGAGGCGAGCTCGCGGCG AAG -3’. MDA-MB-231 shControl or shHIF1A/shHIF2A cells were transfected with the WT or mutant ITAG6-Luc reporter and with or without a plasmid expressing a stabilized version of murine HIF-1α: pcDNA3 mHIF-1α MYC-tag P402A/P577A/N813A (AddGene 44028) using Lipofectamine 2000 (Life Technologies) according to the manufacturer instructions. Forty-eight hours post-transfection, the transfected cells were exposure to hypoxia (0.5 % O_2_) for 6 h. Luciferase activities were measured from lysed cells using the Dual-Luciferase Reporter Assay System (Promega) and normalized to protein concentrations. Relative luciferase activities were calculated by setting the luciferase activity in control cells (shControl cells at normoxia not transfected with mHIF-1α set to 1.0). Data presented are representative of three independent experiments.

### Limiting dilution transplantation and lung metastasis assays using sorted PyMT tumor cells

Four cell inputs (25, 50, 100 or 200 cells) per sorted population (CD49f^+^/CD24^High^ or CD49f^Neg^/CD24^Low^) were injected into the cleared mammary fat pads of 3-wk old FVB/Nj recipients (n ≥6 mice/cohort) in a volume of 10 μl of 1:1 HBSS: growth-factor reduced Matrigel (BD Biosciences, San Jose, CA). Mice were palpated 1-2×/week and tumors measured with digital calipers. Lesions were scored positive for tumor initiation when the diameter of the lesion was ≥ 5 mm in diameter. Data was input into the Extreme Limiting Dilution Analysis (ELDA) software for estimation of TIC frequency, as in [3]. In a subset of mice, tumors were allowed to grow to a size of 500 mm^3^ and the animals were sacrificed to harvest lungs and to compare the mean of lung metastases per population among cohorts as scored by evaluating H&E-stained paraffin sections as in [3].

### Tumorsphere assay

Single cells derived from digested PyMT+ tumors were FACS-sorted based on the expression of CD49f and CD24 into two populations: CD49f^+^/CD24^High^ and CD49f^Neg^/CD24^Low^. Sorted cells from each population were immediately plated post-sorting at a density of 15 cells/μl into ultra low adhesion tissue culture plates (Corning, NY, USA) containing tumorsphere medium and the sphere formation efficiency (SFE) calculated as in [3].

### Invasion assays

MDA-MB-231 cells were serum-starved overnight prior to plating in invasion assays. Trypsinized cells were plated into the upper chamber of control (8 μm pore) or Matrigel-coated Transwell inserts (BD Biosciences) containing serum-free DMEM-Hi medium at a density of 10,000 cells/well and attracted to medium containing 10 % FBS. Cells were allowed to migrate/invade for 24 h at normoxia or hypoxia (0.5 % O_2_) (*n* ≥ 3 wells per genotype per condition). To compare invasion of CD49f-FITC-sorted populations, cells were collected post-sorting into serum-coated FACS tubes as described above, washed once with PBS and then allowed to recover from sorting overnight at 4 °C in tumorsphere stem cell media, as in [3]. Cells were then plated at a density of 30,000−50,000 cells/well and exposed to hypoxia (0.5 % O_2_) for 24 to 48 h (*n* ≥3 wells/population/experiment). Crystal violet stained filters were imaged using ImageJ software and the invasion index calculated following correction for random migration per manufacturer's instructions.

### ITGA6 siRNA knockdown and ectopic ITGA6 expression

To determine whether ITGA6 is necessary for invasion in MDA-MB-231 cells, cells were transiently transfected with Lipofectamine 3000 at 50 % confluence to 100nM of a pool of siRNAs targeting ITGA6 (Dharmacon, SMARTpool, catalog number L-007214) or to a siRNA GFP control described in [78] for 18 h. The next day, cells were exposed to complete growth medium for 8 h, then serum-starved overnight (20–22 h), then prepared the next day for invasion assays as described above. Data shown is the grand mean ± SEM of three independent experiments. For ectopic expression of ITGA6, a pcDNA3.1 plasmid expressing ITGA6 transcript variant 2 (NM_000210; GenEZ ORF clone: OHu24858, GenScript, Piscataway, NJ) was transfected by Lipofectamine 3000 into shHIF1A/shHIF2A MDA-MB-231 cells and a stable cell line was created by selection with neomycin (G418, 1200 ng/ml). Invasion was compared relative to shControl cells transfected with pcDNA3.1-neo empty vector and to parental shHIF1A/shHIF2A MDA-MB-231 cells. Data shown is the grand mean ± SEM of three independent experiments.

### Breast tumor subtype analysis and correlation with survival

Relative *ITGA6* expression among PAM50-classified subtypes was based on normalized data downloaded from The Cancer Genome Atlas (TCGA) website [30]. *p*-values were calculated by ANOVA with Bonferroni correction. Correlation between *HIF1A* or *HIF2A* and *ITGA6* mRNA expression was also analyzed using TCGA data. Correlations between *ITGA6* expression and survival were derived using the Gene Expression Omnibus [GEO: GSE1992] data set [42]. Normalized *ITGA6* expression values were divided into four equal quartiles based on distribution frequencies. Thirty-seven tumors were included in the top quartile (≥75 % percentile) defining “high” *ITGA6* expression (≥0.484), and 45 tumors were included in in the lowest quartile (≤25 % percentile), defining “low” *ITGA6* expression (≤ −0.813). Survival analyses were performed using Prism 5.0 (GraphPad, San Diego, CA) and *p*-values calculated by the log-rank test.

## Additional files

Additional file 1: Figure S1. Expression of HIF-1α and HIF-2α in MDA-MB-231 shRNA cells. A-B. qRT-PCR was performed to evaluate changes in *HIF1A* (B) or *HIF2A* (C) mRNA levels in all 4 genotypes of MDA-MB-231 cells when cultured at 0, 6 or 24 hours of hypoxia (0.5% O_2_). The mean ± SEM relative expression (exp.) of *HIF1A* or *HIF2A* to *PPIA* (cyclophilin A) is shown (*n*=3 biological experiments). **p*-value <0.05 by two-way ANOVA; n.s. equals not significant. C. Western blotting of high-salt enriched, whole cell extracts prepared as in [3] to detect HIF-1α and HIF-2 α proteins in all 4 genotypes of MDA-MB-231 cells cultured at normoxia (N) or hypoxia (H; 0.5 % O_2_, 24h). TBP is included as a loading control. (JPG 432 KB) Additional file 2: Figure S2. A decrease in CD49f^+^ cells is conserved in HIF shHIF1A/shHIF2A MCF-7 cells. Similar to MDA-MB-231 cells, the percentage of luminal, ER+ MCF-7 HIF shHIF1A/shHIF2A cells that express CD49f is reduced as compared to shControl cells (data shown is representative of three independent experiments). (JPG 225 KB) Additional file 3: Figure S3. ChIP data at the -1333 HRE site and additional ChIP assay controls. A. Less than a 50% site fold enrichment was observed at the -1333 putative HRE site as compared with shHIF1A MDA-MB-231 cells. B. ChIP assay controls for MDA-MB-231 cells following IP with (anti-rabbit IgG) antibodies or IP with HIF-1α or HIF-2α at a non-HRE site present in the *ITGA6* promoter. All data represent the mean fold-change ± SEM for technical replicates, and are representative of three independent experiments. C. Deletion of *Hif1a* in PyMT tumor cells exposed to hypoxia for 6 hours reduces enrichment of HIF-1α binding at an HRE located at -1690 in the *Itga6* promoter. As a positive control, HIF-1α binding to a previously characterized functional HRE in the murine *Vegf* promoter was included [79]. All primers and genomic sequence information are reviewed in Additional file 7: Table S4. (JPG 401 KB) Additional file 4: Figure S4. Representative gating strategy and post-sort analysis of mammary tumor cells isolated from MMTV-PyMT+ transgenic mice. Late stage carcinomas derived from MMTV-PyMT+ transgenic female mice were digested to obtain single tumor cells, which were stained with CD49f-FITC, CD24-PE, anti-mouse CD31-biotin and the anti-mouse biotin-conjugated lineage (Lin) panel, detected by SA-APC, and sorted on a BD Biosciences FACSAria cell sorter as in [3]. After gating for cell viability (against 7-AAD+ cells), singlets were enriched based on forward scatter (FSC) profiles, followed by gating against APC^+^ cells (Lin^+^ and/or CD31^+^). Two populations of cells were then collected in a two-way sort: CD49f^+^/CD24^High^ vs. CD49f^Neg^/CD24^Low^. (JPG 535 KB) Additional file 5: Figure S5. Manipulation of ITGA6 expression levels by siRNA knockdown and ectopic expression. (A) (Left) Validation of siRNA knockdown of ITGA6 protein levels in shControl MDA-MB-231 cells following treatment with siRNA; cells exposed to siRNA to GFP are shown as the control. TBP is shown as a loading control. (Right) The levels of *ITGA6* mRNA decrease approximately 10-fold following transfection with the *ITGA6* siRNA SMARTpool. (B) ITGA6 protein expression was compared by western blotting of shControl, shHIF1A/shHIF2A cells and shHIF1A/shHIF2A cells reconstituted with ectopic ITGA6. After correcting for loading based on TBP expression, there was a 37 % percent increase in ITGA6 levels in the shHIF1A/shHIF2A context (quantitated by ImageJ software). (JPG 353 KB) Additional file 6: Figure S6. Representative gating strategy and post-sort analysis of MDA-MB-231 cells sorted by CD49f. shControl cells were cultured to ~80 % confluence and harvested by trypsin-EDTA to prepare single cells for CD49f-FITC staining. Viable cells were gated based on FSC-A vs. SSC-A, followed by gating for singlet events. Gates for CD49f-FITC positive or negative cells were based on the anti-rat isotype antibody control. Two populations of cells were then collected in a two-way sort based on purity, gating for the lowest and the highest (~20–25 % upper or lower) of CD49f-expressing cells: CD49f^High^ vs. CD49f ^Low^. Sort purity and viability (by 7-AAD staining) were confirmed in each sorted population. (JPG 523 KB) Additional file 7: Table S1-S4. Tables related to reagents and primers used in the Methods. (DOCX 24 kb)

## Abbreviations

CAR9: carbonic anhydrase 9
ChIP: chromatin immunoprecipitation
CSC: cancer stem cells
ECM: extracellular matrix
ELDA: extreme limiting dilution analysis
EpCAM: epithelial cell adhesion molecule
EPO: erythropoietin
ER: estrogen receptor
HIF: hypoxia-inducible factor
HRE: hypoxia response element
Ints3: integrator complex subunit 3
ITGA6; CD49f: integrin alpha 6
KO: knockout
Lin: hematopoietic lineage
Luc: luciferase
MBC: metastatic breast cancer
MMTV: mouse mammary tumor virus
OS: overall survival
PHD3: prolyl hydroxylase 3
PPIA: cyclophilin A
PyMT: polyoma virus middle T
RFS: recurrence free survival
SA: streptavidin
SFE: sphere formation efficiency
TCGA: The Cancer Genome Atlas
TIC: tumor initiating cell
WISP2: Wnt-inducible signaling protein 2
