# Supplementary figures and images for: ITGA6 is directly regulated by hypoxia-inducible factors and enriches for cancer stem cell activity and invasion in metastatic breast cancer models
Source: Mol Cancer. 2016 Mar 22;15:26. doi: 10.1186/s12943-016-0510-x (PMC4802728; doi:10.1186/s12943-016-0510-x)

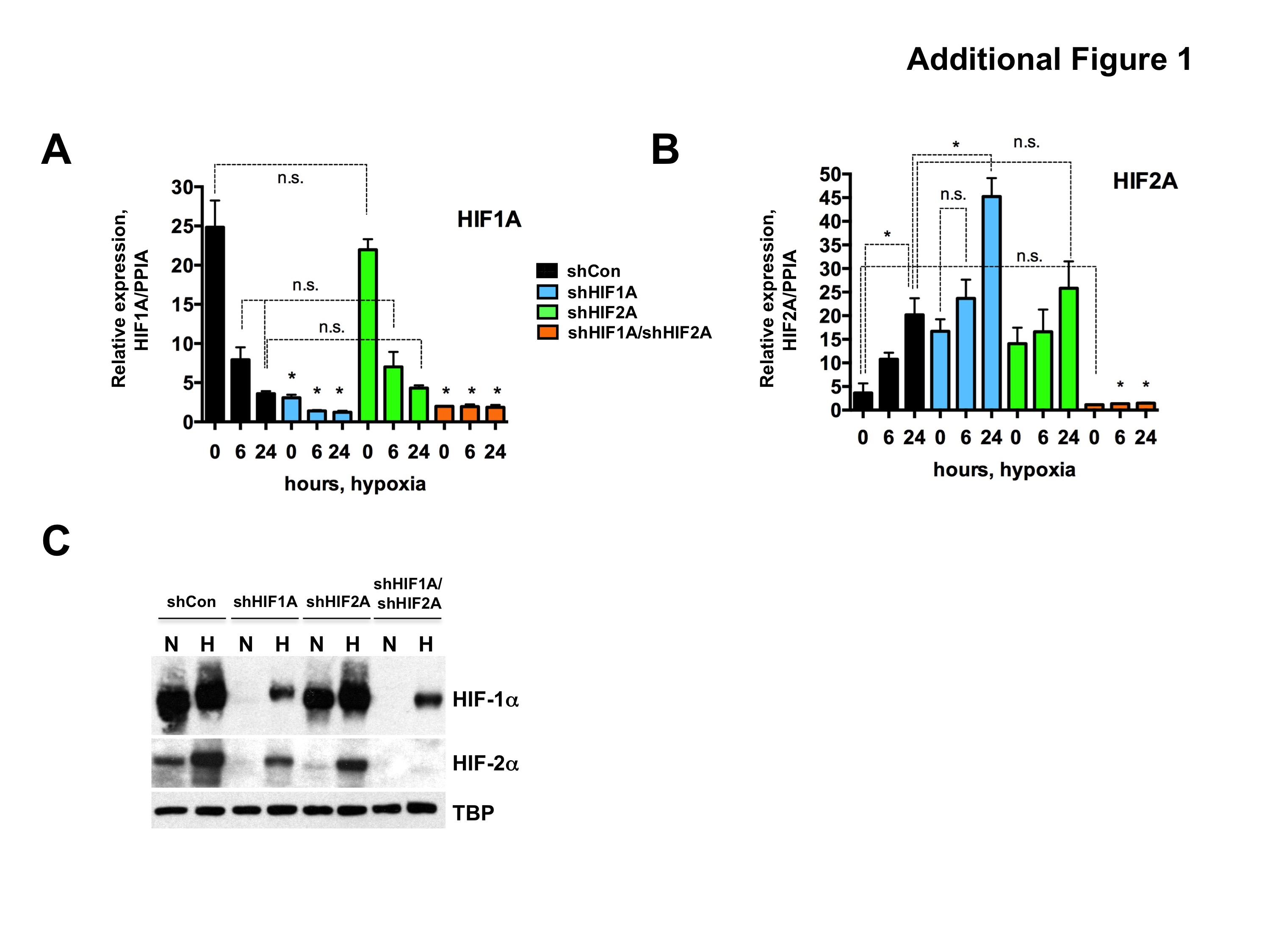

Supplement: Additional file 1: Figure S1. — Expression of HIF-1α and HIF-2α in MDA-MB-231 shRNA cells. A-B. qRT-PCR was performed to evaluate changes in HIF1A (B) or HIF2A (C) mRNA levels in all 4 genotypes of MDA-MB-231 cells when cultured at 0, 6 or 24 hours of hypoxia (0.5% O2). The mean ± SEM relative expression (exp.) of HIF1A or HIF2A to PPIA (cyclophilin A) is shown (n=3 biological experiments). *p-value <0.05 by two-way ANOVA; n.s. equals not significant. C. Western blotting of high-salt enriched, whole cell extracts prepared as in [3] to detect HIF-1α and HIF-2 α proteins in all 4 genotypes of MDA-MB-231 cells cultured at normoxia (N) or hypoxia (H; 0.5 % O2, 24h). TBP is included as a loading control. (JPG 432 KB) [file 12943_2016_510_MOESM1_ESM.jpg]

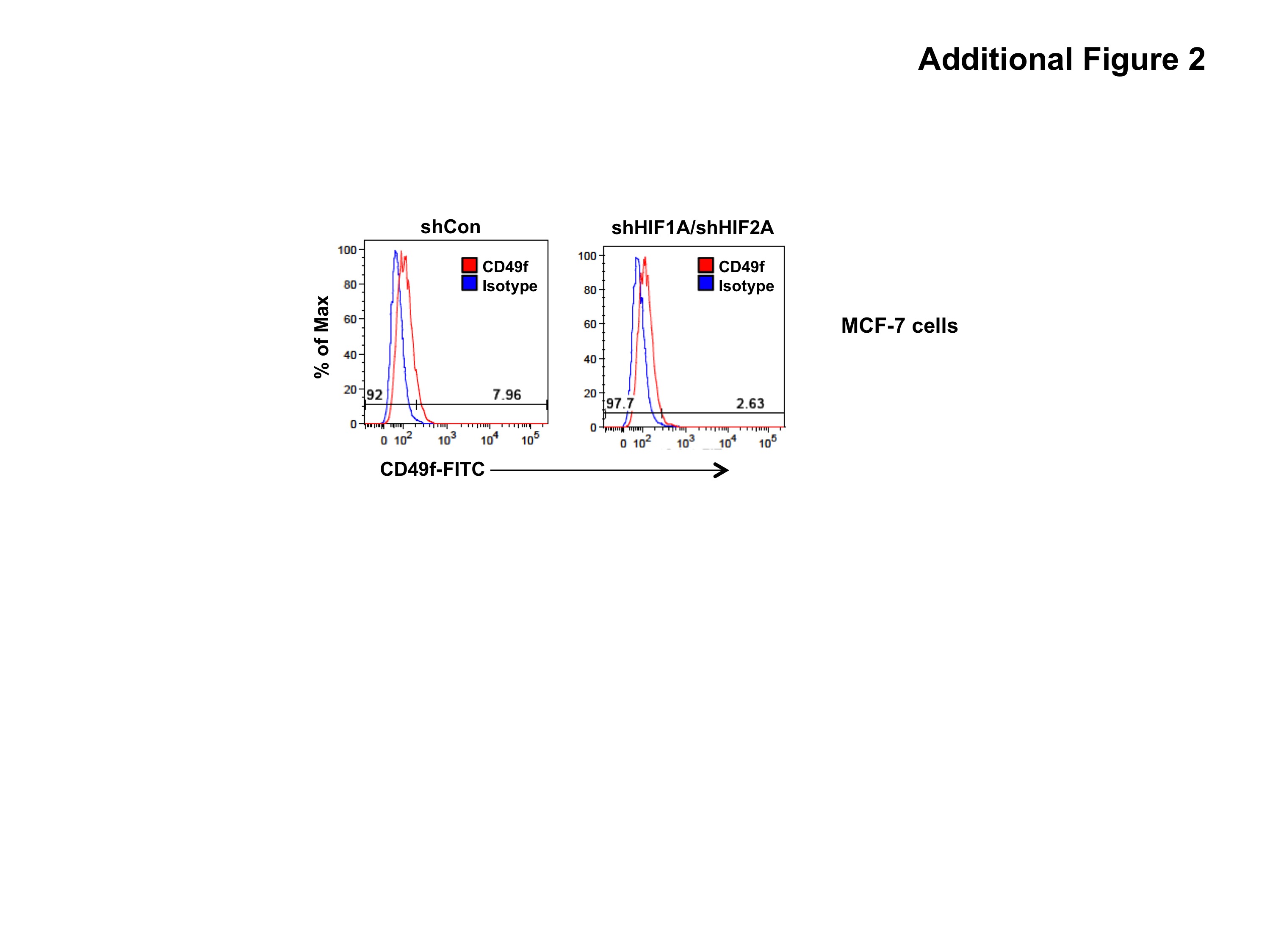

Supplement: Additional file 2: Figure S2. — A decrease in CD49f+ cells is conserved in HIF shHIF1A/shHIF2A MCF-7 cells. Similar to MDA-MB-231 cells, the percentage of luminal, ER+ MCF-7 HIF shHIF1A/shHIF2A cells that express CD49f is reduced as compared to shControl cells (data shown is representative of three independent experiments). (JPG 225 KB) [file 12943_2016_510_MOESM2_ESM.jpg]

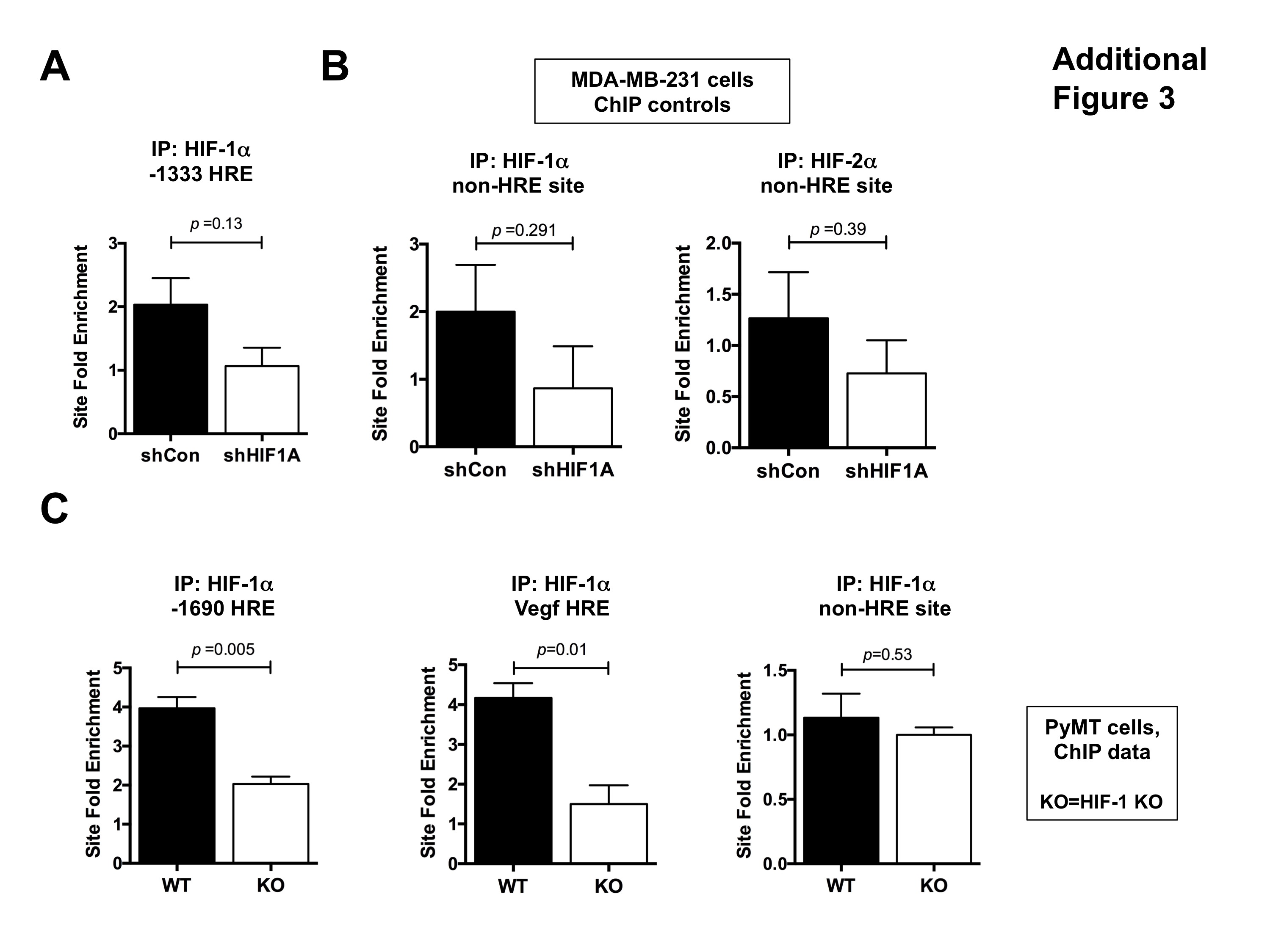

Supplement: Additional file 3: Figure S3. — ChIP data at the -1333 HRE site and additional ChIP assay controls. A. Less than a 50% site fold enrichment was observed at the -1333 putative HRE site as compared with shHIF1A MDA-MB-231 cells. B. ChIP assay controls for MDA-MB-231 cells following IP with (anti-rabbit IgG) antibodies or IP with HIF-1α or HIF-2α at a non-HRE site present in the ITGA6 promoter. All data represent the mean fold-change ± SEM for technical replicates, and are representative of three independent experiments. C. Deletion of Hif1a in PyMT tumor cells exposed to hypoxia for 6 hours reduces enrichment of HIF-1α binding at an HRE located at -1690 in the Itga6 promoter. As a positive control, HIF-1α binding to a previously characterized functional HRE in the murine Vegf promoter was included [79]. All primers and genomic sequence information are reviewed in Additional file 7: Table S4. (JPG 401 KB) [file 12943_2016_510_MOESM3_ESM.jpg]

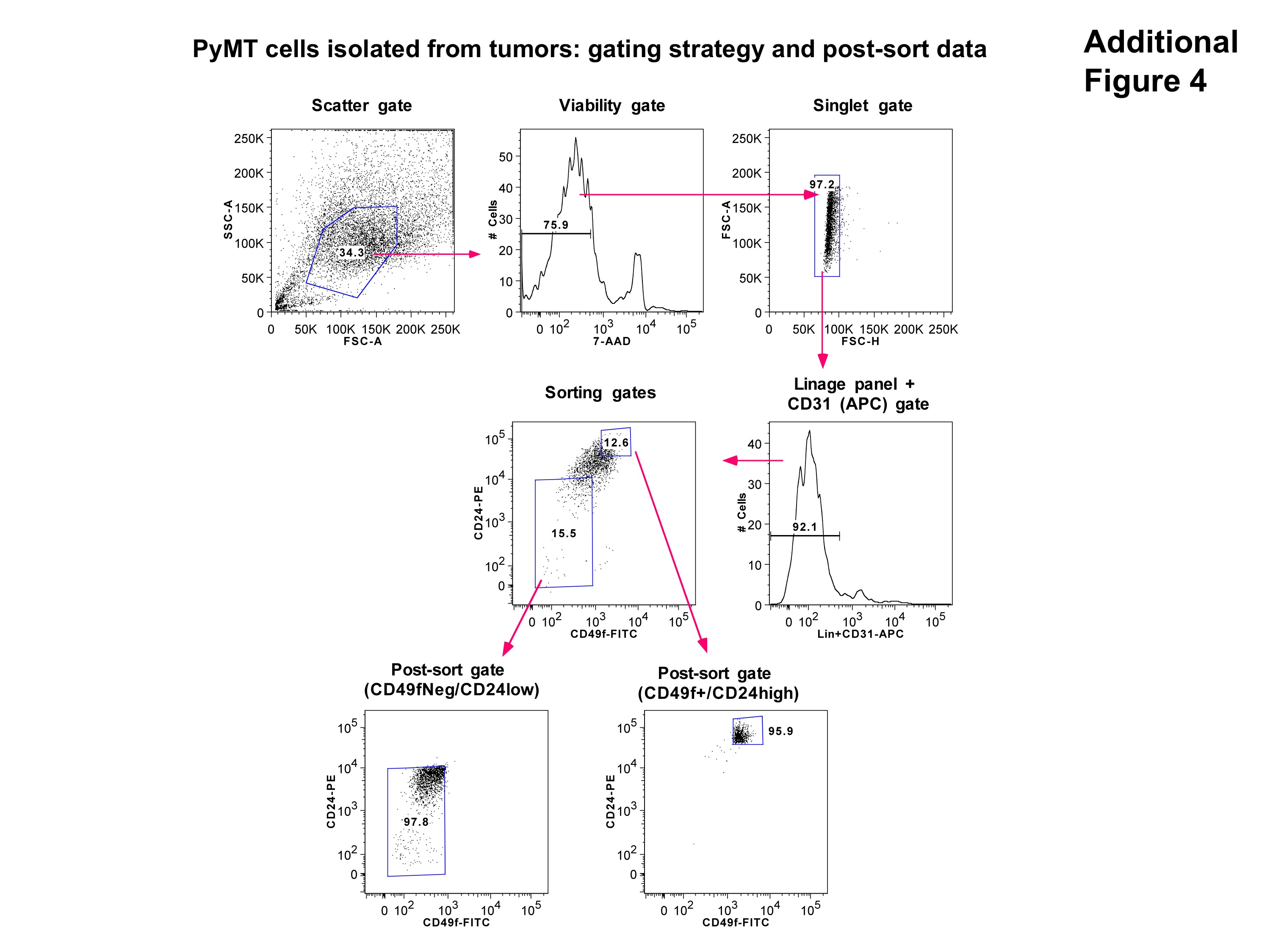

Supplement: Additional file 4: Figure S4. — Representative gating strategy and post-sort analysis of mammary tumor cells isolated from MMTV-PyMT+ transgenic mice. Late stage carcinomas derived from MMTV-PyMT+ transgenic female mice were digested to obtain single tumor cells, which were stained with CD49f-FITC, CD24-PE, anti-mouse CD31-biotin and the anti-mouse biotin-conjugated lineage (Lin) panel, detected by SA-APC, and sorted on a BD Biosciences FACSAria cell sorter as in [3]. After gating for cell viability (against 7-AAD+ cells), singlets were enriched based on forward scatter (FSC) profiles, followed by gating against APC+ cells (Lin+ and/or CD31+). Two populations of cells were then collected in a two-way sort: CD49f+/CD24High vs. CD49fNeg/CD24Low. (JPG 535 KB) [file 12943_2016_510_MOESM4_ESM.jpg]

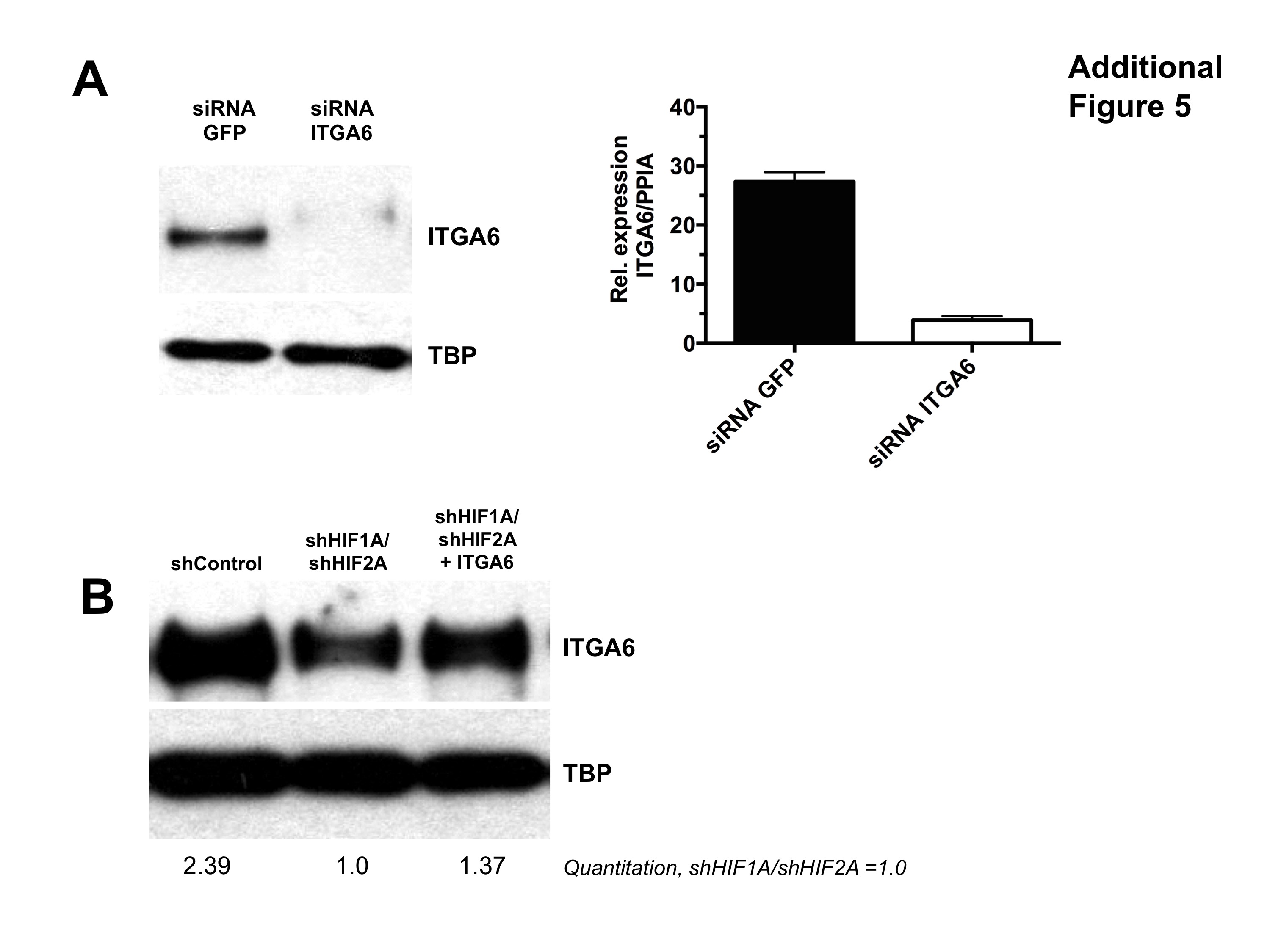

Supplement: Additional file 5: Figure S5. — Manipulation of ITGA6 expression levels by siRNA knockdown and ectopic expression. (A) (Left) Validation of siRNA knockdown of ITGA6 protein levels in shControl MDA-MB-231 cells following treatment with siRNA; cells exposed to siRNA to GFP are shown as the control. TBP is shown as a loading control. (Right) The levels of ITGA6 mRNA decrease approximately 10-fold following transfection with the ITGA6 siRNA SMARTpool. (B) ITGA6 protein expression was compared by western blotting of shControl, shHIF1A/shHIF2A cells and shHIF1A/shHIF2A cells reconstituted with ectopic ITGA6. After correcting for loading based on TBP expression, there was a 37 % percent increase in ITGA6 levels in the shHIF1A/shHIF2A context (quantitated by ImageJ software). (JPG 353 KB) [file 12943_2016_510_MOESM5_ESM.jpg]

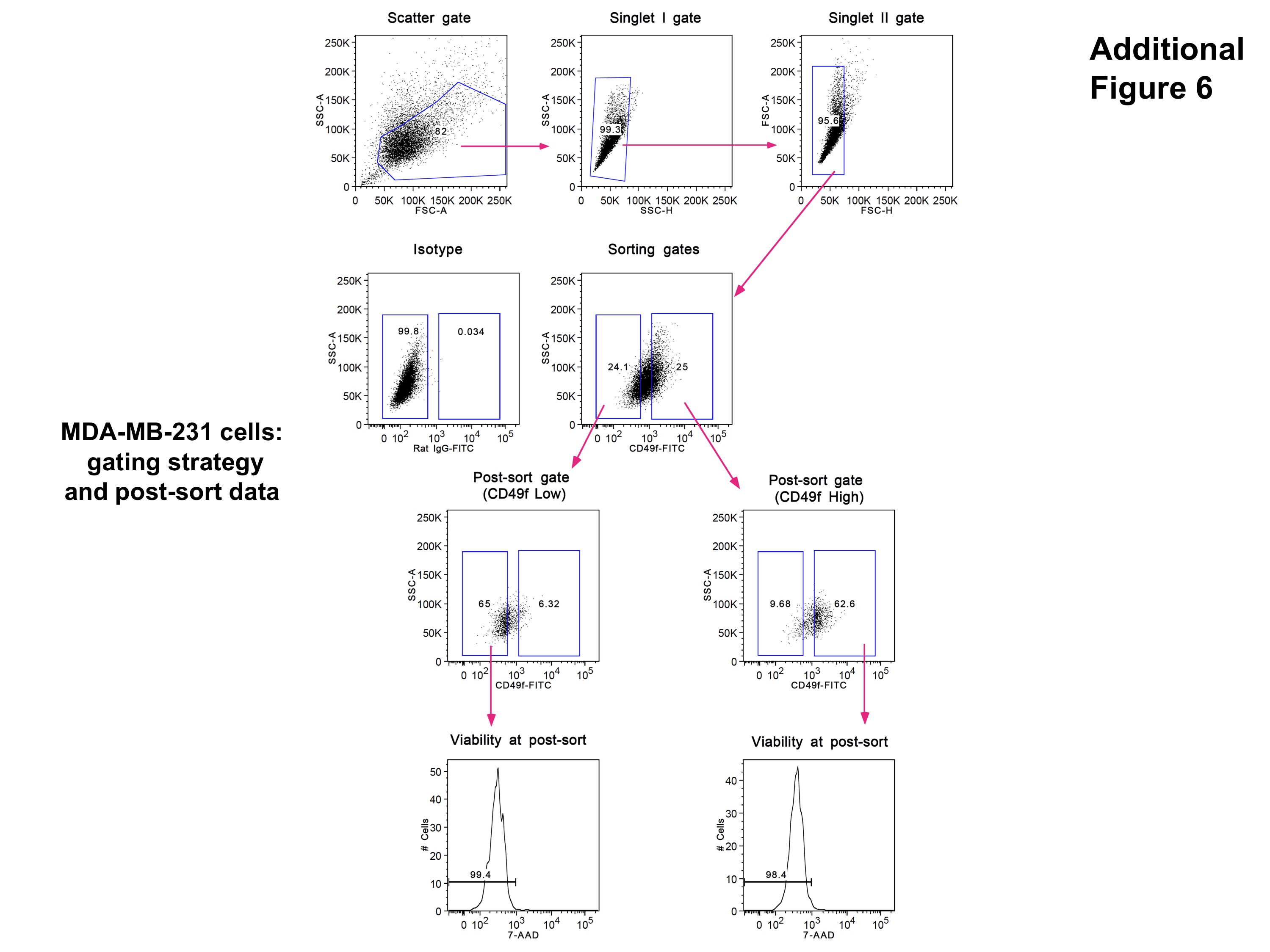

Supplement: Additional file 6: Figure S6. — Representative gating strategy and post-sort analysis of MDA-MB-231 cells sorted by CD49f. shControl cells were cultured to ~80 % confluence and harvested by trypsin-EDTA to prepare single cells for CD49f-FITC staining. Viable cells were gated based on FSC-A vs. SSC-A, followed by gating for singlet events. Gates for CD49f-FITC positive or negative cells were based on the anti-rat isotype antibody control. Two populations of cells were then collected in a two-way sort based on purity, gating for the lowest and the highest (~20–25 % upper or lower) of CD49f-expressing cells: CD49fHigh vs. CD49f Low. Sort purity and viability (by 7-AAD staining) were confirmed in each sorted population. (JPG 523 KB) [file 12943_2016_510_MOESM6_ESM.jpg]
