# Supplementary material for: ITGA6 is directly regulated by hypoxia-inducible factors and enriches for cancer stem cell activity and invasion in metastatic breast cancer models
Source: Mol Cancer. 2016 Mar 22;15:26. doi: 10.1186/s12943-016-0510-x (PMC4802728; doi:10.1186/s12943-016-0510-x)
Supplement: Additional file 7: Table S1-S4. — Tables related to reagents and primers used in the Methods. (DOCX 24 kb) [file 12943_2016_510_MOESM7_ESM.docx]

**Additional file 7** Tables related to reagents and primers used in the Methods.

**Additional Table S1** Antibody source and dilution factors for reagents utilized in FACS analysis, western blotting, ChIP and IF staining assays.

| **Antibody** | **Source (Catalog #)** | **Dilution** | **Purpose** |
| --- | --- | --- | --- |
| anti-mouse/human CD49f-FITC | BD Biosciences (555735) | 1:200  1:50 | FACS  IF |
| anti-mouse CD31-biotin | BD Biosciences (553371) | 1:200 | FACS  PyMT cells |
| anti-mouse CD24-PE | BD Biosciences (553262) | 1:200 | FACS  PyMT cells |
| anti-mouse lineage panel-biotin | BD Biosciences (559971) | 1:200 | FACS  PyMT cells |
| SA-APC | BD Biosciences (554067) | 1:200 | FACS  PyMT cells |
| anti-HIF-1α | Abcam (ab2185) | 1:500 1:150 | IF  ChIP, mouse cells |
| Anti-ITGA6 (α6; rabbit monoclonal) | Life Technologies, Novex (701178) | 1:250 | western blot |
| Anti-TBP | Abcam (ab818) | 1:2,000 | western blot |
| anti-HIF-1α | Santa Cruz (sc-10790X) | 1:150 | ChIP, human cells |
| anti-HIF-2α | Novus Biologicals (100-122) | 1:150 | ChIP |
| anti-rabbit IgG | Cell Signaling (7074P2) | 1:250 | ChIP |

**Additional Table S2** Primers and Roche Universal Probe Library (UPL) FAM-labeled probes utilized in primer and probed-based real-time PCR assays. All assays were designed using the Roche Universal ProbeLibrary Assay Design Center website (<http://qpcr.probefinder.com/organism.jsp>).

| **Gene Symbol** | **NCBI Accession** | **Forward Primer** | **Reverse Primer** | **UPL ID** |
| --- | --- | --- | --- | --- |
| Itga6 | NM_008397 | attcaggagtagcttggtggat | ttctcttgaagaagccacacttc | 79 |
| Ints3 | NM_145540 | gtggctgttattgactctgcac | caggttccccatcatcacat | 17 |
| ITGA6_A or B_  (gene expression assay) | NM_000210 | tttgaagatgggccttatgaa | ccctgagtccaaagaaaaacc | 22 |
| ITGA6_A or B_  (confirm siRNA knockdown) | NM_000210 | Agcctcttcggcttctcg | ttggctctctgcagtggaa | 29 |
| PPIA  (cyclophilin A) | NM_021130 | atgctggacccaacacaaat | tctttcactttgccaaacacc | 48 |

**Additional Table S3** Primers used in SYBR green qRT-PCR reactions to detect human genes.

| **Gene Symbol** | **NBCI Accession** | **Forward Primer** | **Reverse Primer** |
| --- | --- | --- | --- |
| HIF1A | NM_001530 | tccgatggaagcactagaca | tggtgacaactgatcgaagg |
| PPIA | NM_021130 | atgctggacccaacacaaat | tctttcactttgccaaacacc |
| ITGA6_A or B_ | NM_000210 | tttgaagatgggccttatgaa | ccctgagtccaaagaaaaacc |
| PHD3 | NM_022073 | atcgacaggctggtcctcta | cttggcatcccaattcttgt |
| CAR9 | NM_001216 | gggtgtcatctggactgtgtt | cttctgtgctgccttctcatc |
| WISP2 | NM_003881 | ctcctctgcctcctctcaaa | gtctccccttcccgatacag |

**Additional Table S4** Primers used in ChIP assays in human MDA-MB-231 and murine PyMT tumor cells.

| **Genomic Region** | **Sequence**  **Accession** | **ChIP Antibody** | **Forward Primer** | **Reverse Primer** |
| --- | --- | --- | --- | --- |
| -170; *ITGA6* | NM_00210 | HIF-1α or HIF-2α | ctccctcgctctgtgctact | gtctccagctgcccggta |
| -1333; *ITGA6* |  | HIF-1α | acagctcctgctctttcctg | agaaccccatggttttcagc |
| -1762; *ITGA6* |  | HIF-1α or HIF-2α | ggaccccatctctctggat | tgattttggagagcacacagtt |
| -2150; non-HRE *ITGA6* |  | HIF-1α or HIF-2α | gggaagacaggaatcaatgg | tcacaacctggcaaatgaaa |
| -1690; *Itga6* | NM_008397 | HIF-1α | ccagggcaagatgtgagatt | acccaaagcgtccttctaca |
| -1100; non-HRE *Itga6* |  | HIF-1α | caactgtaatttgaaacatctgcaa | tcctctgacatttagattagcatgttt |
| *EPO* HRE, 3’UTR | NM_000799 | HIF-1α or HIF-2α | gctggcctctggctctcatgg | cagggttggcagctgccttactg |
| *Vegfa*, promoter | NM_009505 | HIF-1α | ctggcttcagttccctggcaacatctct | cctggggtgaatgggatcctctgg |
